# The Ecm11-Gmc2 Complex Promotes Synaptonemal Complex Formation through Assembly of Transverse Filaments in Budding Yeast

**DOI:** 10.1371/journal.pgen.1003194

**Published:** 2013-01-10

**Authors:** Neil Humphryes, Wing-Kit Leung, Bilge Argunhan, Yaroslav Terentyev, Martina Dvorackova, Hideo Tsubouchi

**Affiliations:** MRC Genome Damage and Stability Centre, University of Sussex, Brighton, United Kingdom; Stowers Institute for Medical Research, United States of America

## Abstract

During meiosis, homologous chromosomes pair at close proximity to form the synaptonemal complex (SC). This association is mediated by transverse filament proteins that hold the axes of homologous chromosomes together along their entire length. Transverse filament proteins are highly aggregative and can form an aberrant aggregate called the polycomplex that is unassociated with chromosomes. Here, we show that the Ecm11-Gmc2 complex is a novel SC component, functioning to facilitate assembly of the yeast transverse filament protein, Zip1. Ecm11 and Gmc2 initially localize to the synapsis initiation sites, then throughout the synapsed regions of paired homologous chromosomes. The absence of either Ecm11 or Gmc2 substantially compromises the chromosomal assembly of Zip1 as well as polycomplex formation, indicating that the complex is required for extensive Zip1 polymerization. We also show that Ecm11 is SUMOylated in a Gmc2-dependent manner. Remarkably, in the unSUMOylatable *ecm11* mutant, assembly of chromosomal Zip1 remained compromised while polycomplex formation became frequent. We propose that the Ecm11-Gmc2 complex facilitates the assembly of Zip1 and that SUMOylation of Ecm11 is critical for ensuring chromosomal assembly of Zip1, thus suppressing polycomplex formation.

## Author Summary

Meiosis is central to the life cycle of sexually reproducing organisms. The first round of division (meiosis I) is unique to meiosis in that homologous chromosomes are segregated to opposite poles. The tight association between homologous chromosomes is essential for their faithful segregation. To establish such association, meiosis employs a unique, homologous recombination-dependent mechanism that facilitates the recognition, association, and reciprocal exchange of DNA strands of homologous chromosomes, thus providing physical connections between homologous chromosomes. All these events take place in the context of an intricate structure called the synaptonemal complex (SC). Within this complex, the axis of one chromosome is aligned at close proximity with the axis of its homologue. This alignment stretches along the entire length of the chromosome pair, with zipper-like structures, called transverse filaments, holding axes together. In this work, we identified the Ecm11-Gmc2 complex as a novel component of the SC, promoting the assembly of transverse filaments. Importantly, we demonstrate that post-translational modification of Ecm11 with SUMO (small ubiquitin-like modifier) is critical for ensuring the chromosomal loading of transverse filaments. Thus, our work provides a molecular basis for how homologous chromosomes become tightly associated during meiotic prophase.

## Introduction

Meiosis is a special type of cell cycle necessary for sexual reproduction [1]. During meiosis, a diploid cell undergoes one round of DNA replication followed by two rounds of successive nuclear segregation, meiosis I and meiosis II respectively. At meiosis I, homologous chromosomes are segregated to opposite poles whereas at meiosis II, sister chromatids separate. As a result, four haploid gametes form from one diploid progenitor cell.

In many organisms, homologous recombination plays two critical roles in ensuring the faithful segregation of homologous chromosomes at meiosis I [2]. First, in early prophase I, homologous recombination provides a means for chromosomes to find their homologous partners, thus facilitating pairing of homologous chromosomes. Second, crossover recombination events provide a physical connection that maintains homologous associations until chromosomes are properly aligned on the metaphase I spindle.

Homologous recombination is concurrent with the dynamic morphological changes of chromosomes. Sister chromatids condense to form chromosome axes, and sister chromatid axes of homologs are juxtaposed at close proximity along their entire lengths, with a proteinaceous transverse filament structure situated in between. This meiosis-specific chromosomal structure is called the synaptonemal complex (SC).

In budding yeast, the Zip1 protein serves as the transverse filament [3]. The deposition of Zip1 occurs progressively, starting at either centromeres or future crossover sites on chromosome arms [4], [5]. The initiation of homologous recombination is a prerequisite for Zip1 polymerization along chromosomes. In the absence of meiotic recombination (e.g., in the *spo11* background where no meiotic double-strand breaks (DSBs) occur), SC components form an aggregate, called the polycomplex, which is not associated with chromosomes [6]. The initiation of Zip1 polymerization also requires a group of proteins belonging to the synapsis initiation complex (SIC), namely Zip2, 3, 4 and Spo16 [6]–[10]. The absence of these proteins leads to a great reduction in Zip1 loading between homologous chromosomes, usually with a high incidence of polycomplex formation.

The SC is important for the control of meiotic recombination. Typically, mutations in genes encoding SIC components or Zip1 disrupt the close association of homologous chromosomes, reduce interhomolog crossing over and alter the pattern of crossover deposition along paired chromosomes [11], [12]. On the other hand, defects in homologous recombination lead to abnormal morphogenesis of the SC [13]–[16]. These observations provide evidence for the close relationship between homologous recombination and SC formation.

Zip1 also plays a distinct role at centromeres. Zip1 loading at a centromere is independent of the initiation of homologous recombination and SIC components. Zip1 functions at the centromere to associate two centromeres of either homologous or non-homologous chromosomes, possibly facilitating the recognition of homologous chromosomes [4]. Furthermore, centromeres serve as potential synapsis initiation sites [17], the timing of which is coordinated by Fpr3 and Zip3, so that meiotic recombination precedes SC formation [18]. The centromere association formed between non-homologous chromosomes is corrected to establish associations between homologous chromosomes as prophase I progresses, and this regulation employs the phosphorylation of Zip1. This phosphorylation is controlled by the DNA damage checkpoint kinase Mec1 and protein phosphatase 4 [19]. Centromeres play an important role at meiosis I, especially when homologs fail to form a crossover [20]. Zip1 stays at centromeres throughout meiosis I, promoting proper chromosome segregation by directly mediating centromere associations [21], [22].

The small ubiquitin-related modifier (SUMO) protein plays an important role in controlling SC formation [23]. First, Zip3, a component of the SIC, has SUMO E3 ligase activity [24]. Second, Zip1 colocalizes with SUMO, both on chromosomes and at the polycomplex, and interacts with SUMO-conjugated proteins [24], [25]. Third, Red1, a major component of meiotic chromosome axes, interacts with SUMO, and is SUMOylated in a Zip3-dependent manner [26], [27]. However, SUMO-decorated SC assembles in the absence of Zip3 (although its extent is diminished relative to wild type), and little SUMO is detectable on chromosome axes in the absence of Zip1 despite the presence of Red1. Thus the mechanism of SUMOylation of SC central region and the precise role of SUMO in mediating homologous synapsis have remained somewhat mysterious.

In this work, we have identified Ecm11 and Gmc2 as novel SC components. They localize initially to synapsis initiation sites, and then to synapsed regions of meiotic chromosomes with extensive overlap with the Zip1 protein. The absence of these proteins compromises the assembly of Zip1 into SC central region and into polycomplex. Furthermore, Ecm11 is SUMOylated in a Gmc2-dependent manner. This SUMOylation is also partly dependent on Zip1 and the SIC components Zip2, Zip4 and Spo16. The SUMOylation of Ecm11 is essential for the proper assembly of Zip1, which is crucial for proper chromosome synapsis in meiosis. Unexpectedly, polycomplex formation became frequent in the SUMOylation-negative *ecm11* mutant. We propose that the Ecm11-Gmc2 complex promotes SC formation by facilitating the assembly of Zip1 and that SUMOylation of Ecm11 is critical for promoting assembly of Zip1 on chromosomes, thus suppressing polycomplex formation.

## Results

### Cytology-based screening identified Ecm11 and Gmc2 as novel components of the synaptonemal complex

Genes important for meiotic recombination tend to be upregulated during the early stages of prophase I, and proteins directly involved in meiotic recombination tend to associate strongly with meiotic chromosomes, showing distinct localization patterns, typically observed as foci or lines on meiotic chromosomes (e.g., [3], [6], [8]). In order to identify proteins potentially involved in meiotic recombination, the localization patterns of proteins encoded by poorly characterized genes whose transcripts are upregulated during early prophase I were systematically examined on spread chromosomes (Materials and Methods). The screening identified two genes, *ECM11* and *GMC2*. The encoded proteins contain domains highly likely to form coiled-coil structures; the C-terminal region, from amino acids 250 to 300 in Ecm11, and two regions from the middle toward the C-terminus, from 100 to 140 and from 160 to 188 respectively, in Gmc2. No obvious orthologs of these proteins have been found in other organisms. Both Ecm11 and Gmc2 show a line-shaped staining pattern throughout the length of paired pachytene chromosomes, reminiscent of the staining pattern of Zip1. *ECM11* was originally proposed to be involved in cell surface biosynthesis based on sensitivity of the null mutant to calcofluor white [28]. In our study, however, we found no evidence that the *ecm11* mutation enhances sensitivity to calcofluor white (Figure S1). Previous reports characterised the *ECM11* gene as a positive effector of meiosis [29], [30]. A recent report identified *GMC2* as a gene important for meiotic recombination and/or SC formation [30]. The molecular functions of these genes, however, remain unclear.

### Ecm11 and Gmc2 are important for crossing over

**Figure 1:**
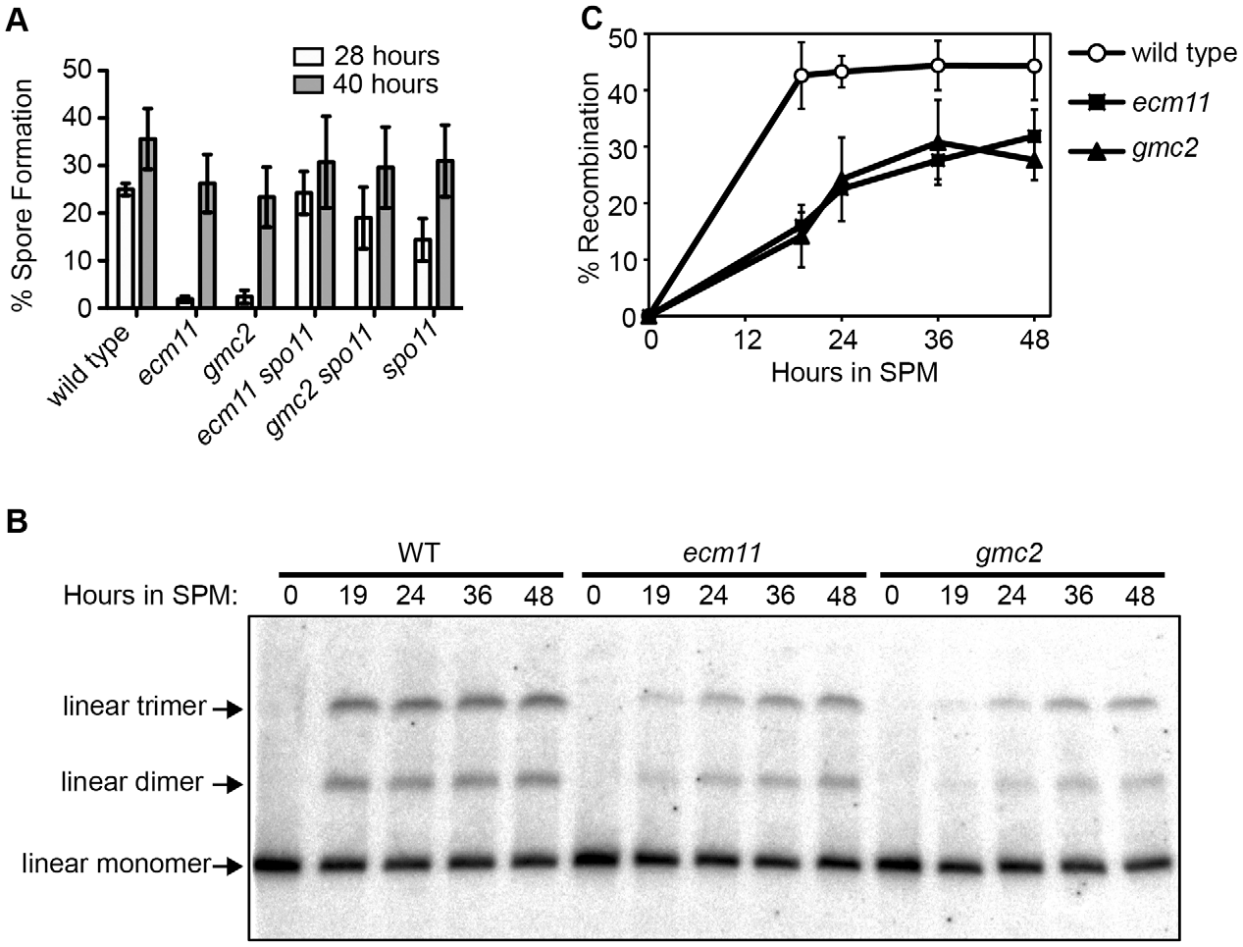
Ecm11 and Gmc2 are required for efficient meiotic crossing over. (A) Sporulation is delayed in the *ecm11* and *gmc2* mutants. Diploid cells were introduced into meiosis and spore formation was examined at indicated time points. (B) Crossing over is reduced in the *ecm11* and *gmc2* mutants. Diploid cells carrying one circular and one linear chromosome III were introduced into meiosis and crossing over between these chromosomes was measured by Southern blotting. The amount of linear dimer and trimer chromosomes represents the efficiency of crossing over. (C) Quantification of crossover products shown in (B). The amount of recombinants was expressed as the ratio (percentage) of the combined signal of linear dimer and trimer bands per the sum of linear monomer, dimer and trimer bands. Error bars represent SEM.

To understand the role of *ECM11* and *GMC2*, the entire ORF of these genes were deleted and the phenotypes were examined. Consistent with previous reports, we found that meiotic cell cycle progression was substantially delayed to a similar level in *ecm11*, *gmc2* and *ecm11 gmc2* double mutants (Figure 1A, Figure S2) [29], [30]. Although sporulation was delayed, the resultant tetrads showed relatively high spore viability; 88%, 76% and 87% of spores were viable in *ecm11*, *gmc2*, and *ecm11 gmc2* double mutant respectively, compared to 98% in wild type (Table 1). Spore viability of the *ecm11* mutant reported previously is much lower (51%) than that of our strain [29]. The reason has been unclear: the difference could be due to the different strain backgrounds used (SK1 in [29] versus BR1919-8B in this work). The cell cycle delay was bypassed by introducing the *spo11* mutation, suggesting that the cause of the cell cycle delay is associated with a defect in meiotic recombination (Figure 1A). A similar bypass effect was reported for the *gmc2* mutant [30]. These observations prompted us to examine the effect of these mutations on meiotic recombination directly. Crossing over was assayed physically in diploid strains carrying one linear and one circular copy of chromosome III. A single crossover between one linear and one circular chromatid results in the production of a linear dimer. A double crossover involving one linear chromatid and both chromatids of the circular chromosome generates a linear trimer. The linear monomers, dimers, and trimers can be separated by pulsed-field gel electrophoresis. In wild type, the level of crossovers plateaued at ∼45% by 19 hours, while only ∼30% was observed at 36 hours in *ecm11* and *gmc2* mutants, showing that crossing over is both delayed and reduced in these mutants (Figure 1B, 1C). Therefore, both Ecm11 and Gcm2 are important for meiotic crossing over.

**Table 1:** Spore viability.

Genotype	Spore viability (%)	Viable/total spores
Wild type (TBR2065)	98	235/240
*ecm11* (TBR4246)	88	351/400
*gmc2* (TBR4327)	76	244/320
*ecm11 gmc2* (TBR4757)	87	139/160

### Ecm11 and Gmc2 are necessary for the efficient assembly of Zip1 in both chromosomes and the polycomplex

**Figure 2:**
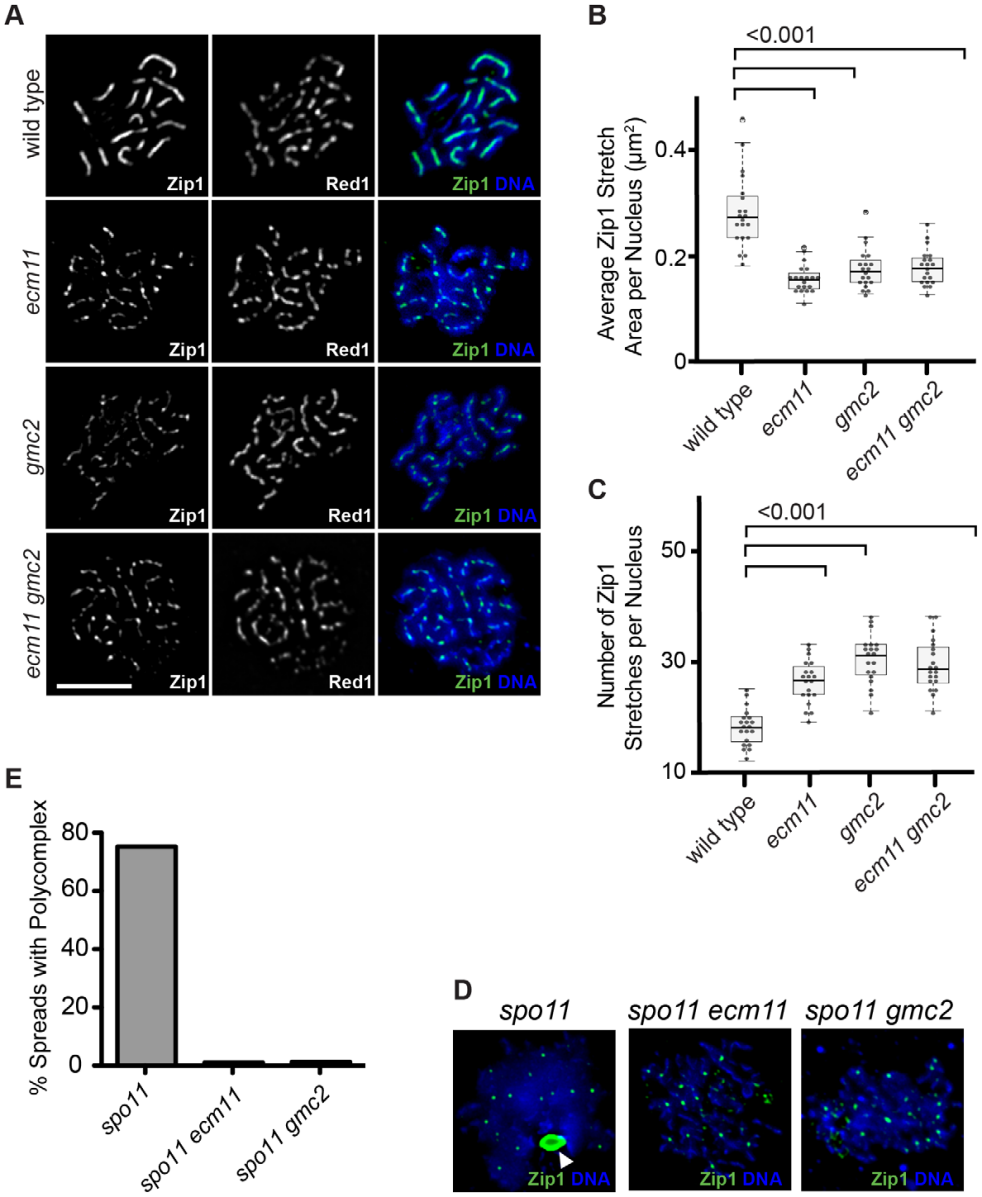
Ecm11 and Gmc2 are necessary for the efficient assembly of Zip1 on chromosomes and in the polycomplex. (A) Zip1 localization is discontinuous on meiotic prophase chromosomes in the *ecm11* and *gmc2* mutants. The localization of Zip1 along with Red1, a component of meiotic chromosome axes, was examined on spread chromosomes. Bar, 5 µm. (B, C) Quantitative analysis of the Zip1 localization. Zip1 stretch area represents the size of one continuous Zip1 staining. See Results and Materials and Methods for more details. (D) Polycomplex formation was abolished in *ecm11* and *gmc2* mutants. Zip1 localization was examined in the *spo11* mutant background. The white arrowhead indicates the location of the polycomplex. Small speckle-like Zip1 staining likely represents the centromeric localization of Zip1. Chromosome spreads were prepared using cells at 20 hours after introduction into meiosis when, in wild type, cells at the pachytene stage are enriched. (E) Quantification of spread nuclei exhibiting a polycomplex.

The apparent similarity of the localization patterns between Ecm11, Gmc2 and Zip1 prompted us to examine a potential role for Ecm11 or Gmc2 in the assembly of Zip1. Meiotic cells were surface spread and the localization of Zip1 was detected by immunostaining. In wild type, Zip1 shows a linear confluent staining pattern throughout the length of paired chromosomes from mid to late prophase I. However, in the absence of either/both Ecm11 and Gmc2, the localization of Zip1 becomes rather discontinuous (Figure 2A). This effect was analyzed quantitatively. The area showing continuous Zip1 staining, defined as Zip1 stretch area, became much smaller in the absence of either/both Ecm11 and Gmc2 (Figure 2B). Correspondingly, the number of Zip1 stretches became higher (Figure 2C). These results strongly suggest that both Ecm11 and Gmc2 are important for the efficient assembly of Zip1 onto chromosomes. The similarity of the phenotypes between each single mutant and the double mutant suggests that Ecm11 and Gmc2 function in the same pathway.

In the absence of Spo11, initiation of meiotic recombination does not occur and SC components form an aggregate called the polycomplex, which is not associated with chromosomes (Figure 2D). A major component of the polycomplex is Zip1. Even in the polycomplex, Zip1 is thought to maintain a highly ordered structure similar to that found in the context of the SC [31]. In the *spo11* mutant, a polycomplex was found in almost 80% of spread chromosomes (Figure 2D, 2E). Strikingly, polycomplex formation was almost completely abolished in the absence of Ecm11 or Gmc2. These results further support the role of Ecm11 and Gmc2 in facilitating the assembly of Zip1.

Zip1 is known to have a function independent of homologous recombination, which is to associate two chromosomes together via their centromeres, or “centromere coupling” [4]. The involvement of Ecm11 and Gmc2 in this aspect of Zip1 function was examined. Centromere coupling can be assessed by visualizing centromeres using a *spo11* diploid. Ctf19, a component of the yeast kinetochore, was tagged with the myc epitope to identify the location of centromeres. A diploid budding yeast cell contains 32 chromosomes representing 16 pairs of homologs. In the *spo11* mutant, ∼18 centromere foci were observed on average whereas the number went up to ∼30 in the *spo11 zip1* mutant, consistent with the involvement of Zip1 in centromere coupling as reported previously (Figure S3) [4]. The absence of Ecm11 or Gmc2 has little effect, if any, on the number of centromere foci detected, ∼20 and ∼19 respectively, suggesting that they are dispensable for centromere coupling.

### Ecm11 is SUMOylated in a Gmc2-dependent manner, and the SUMOylation partially requires Zip1 and some components of the synapsis initiation complex

**Figure 3:**
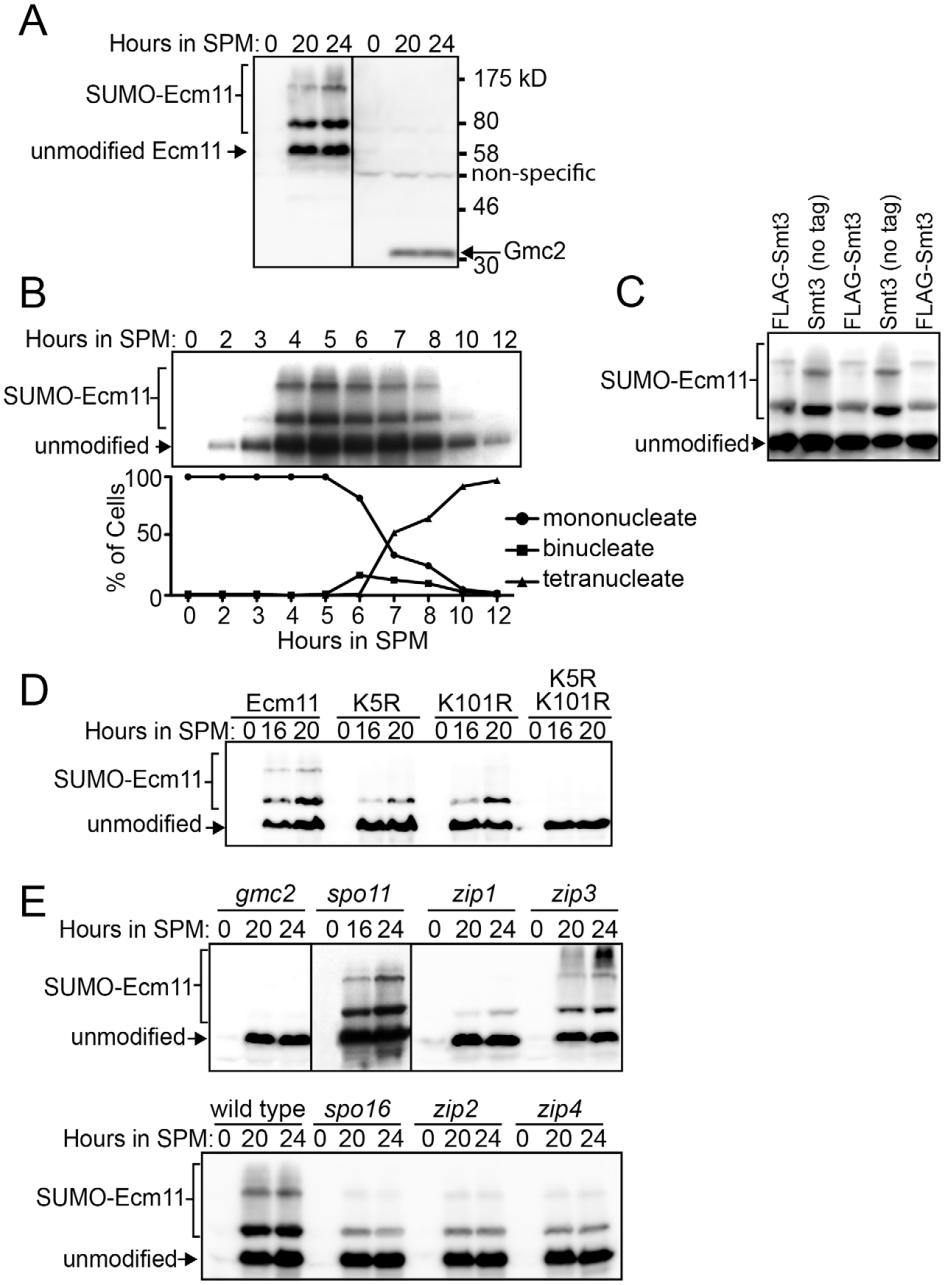
Ecm11 SUMOylation depends on Gmc2 and also partially on Zip1, Zip2, Zip4, and Spo16. (A) Ecm11 and Gmc2 proteins are expressed specifically during meiosis. Diploid strains carrying *ECM11-myc* (left) or *myc-GMC2* (right) were introduced into meiosis and Ecm11 and Gmc2 proteins were detected using anti-myc antibodies by Western blotting. *ndt80* deletion strains were employed. (B) The production and modification of Ecm11 is most abundant before the meiosis I nuclear division. The diploid SK1 strain carrying *ECM11-myc* was introduced into meiosis and samples were taken at indicated time points. Meiotic cell cycle progression was monitored simultaneously by DAPI staining. (C) Ecm11 is SUMOylated. The diploid *ECM11-myc ndt80* strain with FLAG-tagged *SMT3* (FLAG-Smt3) or with untagged *SMT3* (no tag) was introduced into meiosis, samples were taken at 20 hours, and the Ecm11 protein was detected using anti-myc antibodies by Western blotting. FLAG-Smt3 and Smt3 (no tag) samples were loaded alternately to highlight the mobility differences of the modified forms of Ecm11. (D) Both Lysine 5 and 101 are important for SUMOylation of Ecm11. The *ndt80* diploid strain carrying the wild type *ECM11-myc* or its mutated derivatives, *ecm11-K5R*, *ecm11-K101R* or *ecm11-K5R*, *K101R* was introduced into meiosis and samples were taken at indicated time points. The Ecm11 protein was detected using anti-myc antibody by Western blotting. (E) Genetic requirement for the SUMOylation of Ecm11. The diploid *ECM11-myc ndt80* strains carrying homozygous null mutations as indicated were introduced into meiosis and the Ecm11 protein was detected using anti-myc antibody by Western blotting. See Figure S4A, S4B and S4C for Ponceau S staining (loading control) of Western blots shown in (A, B and D).

To further investigate the protein behavior of Ecm11 and Gmc2, these proteins were tagged with the myc epitope (see Materials and Methods). Using a diploid homozygous for either *ECM11-myc* or *myc-GMC2*, these proteins were detected by Western blotting. We employed the *ndt80* mutant background to arrest the meiotic cell cycle at prophase I, since the strain background we used (BR1919-8b background) does not support efficient synchronous entry into meiosis. Consistent with the behavior of the transcripts of the *ECM11* and *GMC2* genes, both proteins were produced specifically during meiosis (Figure 3A). Furthermore, the Ecm11 protein was detected as multiple bands including three major bands, suggesting that Ecm11 is post-translationally modified. To rule out the possibility that this modification is specific to the *ndt80* background, we employed the SK1 strain background in which cells can be synchronously introduced into meiosis. Essentially, the same migration pattern of Ecm11 was obtained in the SK1 background (Figure 3B). The amount of the protein peaked at around 5 hours, which is right before the nuclear division of meiosis I, consistent with the idea that Ecm11 functions during meiotic prophase I.

Zavec et al. (2008) presented some evidence that Ecm11 is SUMOylated during meiosis. We confirmed this idea by attaching three copies of the FLAG epitope at the N-terminus of the Smt3 protein, the budding yeast SUMO protein, in strains containing *ECM11-myc*. The addition of FLAG makes the molecular weight of Smt3 higher, thus a protein covalently attached with FLAG-Smt3 should migrate more slowly in SDS-PAGE than one with untagged Smt3. Indeed, the top two bands were observed with reduced mobility specifically when Smt3 was tagged with FLAG while the bottom band did not change position (Figure 3C), arguing that the upper two bands are SUMOylated while the bottom is not. To further verify the entity of these slow migrating bands, the whole cell extract from cells carrying Ecm11-myc and FLAG-Smt3 were used to immunoprecipitate Ecm11 and the immunoprecipitates were examined for the presence of Ecm11 and SUMO by using anti-FLAG and anti-myc antibodies. Only the two bands that slowed down the migration in Figure 3C were detected with anti-FLAG antibodies (Figure S4E), providing further evidence that Ecm11 is SUMOylated.

There are two canonical SUMOylation target sites within Ecm11, at Lysine 5 (K5) and Lysine101 (K101) [32]. To examine the importance of these sites, they were mutated to Arginine (R) (hereafter referred to as K5R and K101R respectively) and their effect on Ecm11 modification was examined (Figure 3D). Neither K5R nor K101R completely abolished SUMOylation; residual SUMOylation was detected in both K5R and K101R mutants. However, SUMOylation was completely abolished when these two sites were simultaneously mutated, suggesting that both of these sites are modified by SUMOylation. We also mutated the Lysine 5 and 101 to Asparagine [32]. These mutants behaved essentially the same as K5R and K101R (Figure S4D), further supporting the idea that both K5 and K101 are SUMOylated.

Next we examined the role of other synapsis proteins in the SUMOylation process of Ecm11 (Figure 3E). The bands associated with SUMOylation were barely detectable in the absence of Gmc2. A level of SUMOylation comparable to that of wild type was seen in the *spo11* mutant, indicating that the initiation of meiotic recombination is not necessary for Ecm11 SUMOylation. Interestingly, the SUMOylation became less efficient in the absence of Zip1. Next we examined components of the SIC: Zip2, Zip3, Zip4 and Spo16. A substantial reduction in SUMOylation was found in the *zip2*, *zip4* and *spo16* mutants. In *zip3*, no prominent reduction was observed; instead, slower-migrating species than the slowest of the three major bands appeared darker than in wild type, suggesting that Ecm11 might be more extensively modified in *zip3*.

### Ecm11 and Gmc2 are initially recruited to synapsis initiation sites, then deposited between paired homologs in a Zip1-dependent manner

**Figure 4:**
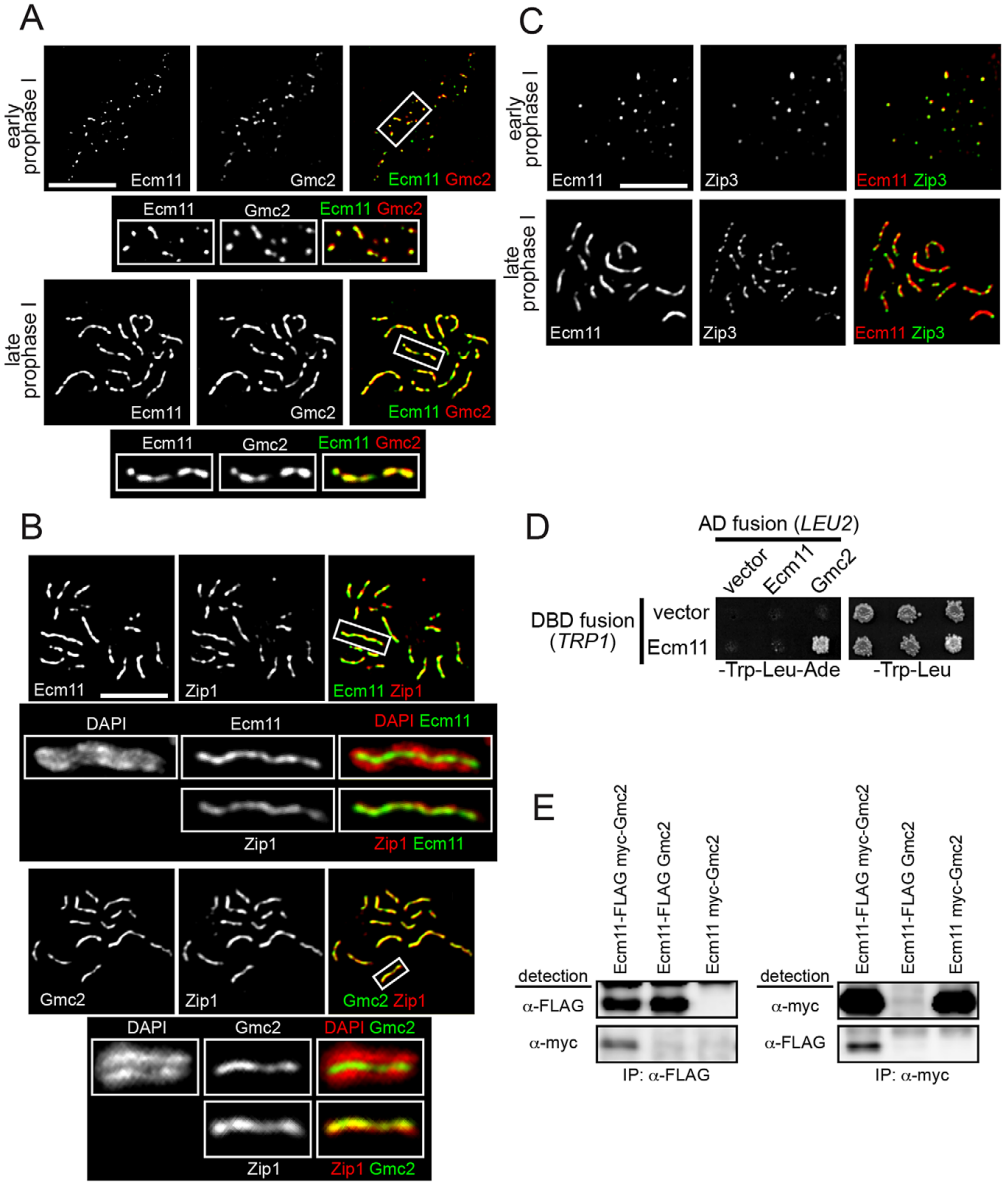
Ecm11 and Gmc2 associate with each other and are localized to the synapsis initiation sites at early prophase I and later to the SC central region. (A) Ecm11 and Gmc2 colocalize throughout meiotic prophase I. A diploid strain carrying *ECM11-FLAG* and *myc-GMC*2 was introduced into meiosis, chromosomes were surface spread and Ecm11 and Gmc2 were immunostained with anti-FLAG and anti-myc antibodies. The boxed areas were magnified and shown below. (B) Both Ecm11 and Gmc2 colocalize with Zip1, and are localized to the area between paired homologs. A diploid strain carrying either *ECM11-myc* or *myc-GMC2* was introduced into meiosis and spread chromosomes were immunostained for Ecm11 (top), Gmc2 (bottom) and Zip1. The boxed areas were magnified and shown below, with either the Ecm11 or Gmc2 localization along with the DAPI or Zip1 staining. (C) Ecm11 colocalizes with Zip3 at early prophase I. A diploid strain carrying *ECM11-FLAG* and *ZIP3-myc* was introduced into meiosis and spread chromosomes were stained for Ecm11 and Zip3 using anti-FLAG and anti-myc antibodies. Bar, 5 µm in (A–C). (D) Ecm11 and Gmc2 show interaction using the yeast two hybrid system. Medium lacking tryptophan and leucine was used to maintain the DBD fusion plasmid (marked with *TRP1*) and the AD fusion plasmid (marked with *LEU2*). Growth on medium lacking adenine (shown on the left) reflects the expression level of the *GAL4-ADE2* reporter gene and is thus a measure of the interaction between two fusion proteins. Shown on the right is growth on medium lacking tryptophan and leucine as a reference. (E) Ecm11 and Gmc2 show interaction in the co-immunoprecipitation assay. Extracts from meiotic cells with both Ecm11 and Gmc2 tagged, only Ecm11 tagged with untagged Gmc2, or only Gmc2 tagged with untagged Ecm11, were subjected to immunoprecipitation experiments with antibodies shown. Immunoprecipitates were analysed by Western blotting with antibodies as indicated.

To obtain further insight into the function of the Ecm11 and Gmc2 proteins, their meiotic localization was examined. They showed extensive colocalization throughout meiotic prophase I; both proteins initially appeared as foci at early prophase I, before forming a line-like staining pattern at late prophase I (Figure 4A, Figure S5A). At pachytene, they showed extensive colocalization with Zip1, positioned continuously between paired homologs along their length (Figure 4B, Figure S5A). Next, to examine the relationship between Ecm11-Gmc2 and synapsis initiation sites, Ecm11-Gmc2 were co-immunostained with Zip3. At early prophase I, extensive colocalization was found between Ecm11 and Zip3 (Figure 4C). Of all the foci carrying Zip3 and/or Ecm11, 17.3% of foci contained Zip3 but not Ecm11 whereas only 5.4% of foci contained Ecm11 but not Zip3 (220 foci were counted). This observation supports the idea that Ecm11 is recruited after the chromosomal localization of Zip3. At later stages, Ecm11 became linear while Zip3 remained as punctate foci (Figure 4C).

Similar localization behavior of Ecm11 and Gmc2 on meiotic chromosomes, along with phenotypic similarities in meiotic recombination and the effect on Zip1 assembly, strongly suggests that Ecm11 and Gmc2 function in the same pathway, possibly as part of the same complex. To directly address this point, a potential interaction between Ecm11 and Gmc2 was examined using yeast two hybrid analysis (Y2H). Full-length Ecm11 was fused to the Gal4-DNA-binding domain (DBD) and full length Ecm11 and Gmc2 to the Gal4 transcription activation domain (AD). These fusion plasmids were used to test for interactions between proteins. Ecm11 and Gmc2 showed a strong interaction (Figure 4D). Gmc2-DBD fusion constitutively activated the reporter by itself, thus was not included for the interaction assay. To further obtain *in vivo* evidence for the interaction between Ecm11 and Gmc2, these proteins were immunoprecipitated from meiotic cell extracts and the precipitates were analyzed by Western blotting. When Ecm11-FLAG was immunoprecipitated with anti-FLAG antibodies, myc-Gmc2 was also present in the precipitate. The reverse is also true; when myc-Gmc2 was immunoprecipitated with anti-myc antibodies, Ecm11-FLAG was precipitated as well. The coimmunoprecipitation of Gmc2 and Ecm11 was resistant to nuclease treatment (Materials and Methods), arguing against the possibility that these proteins are only associated indirectly through DNA. Overall, the Y2H and coimmunoprecipitation assays strongly suggest that Ecm11 and Gmc2 are part of the same protein complex. The strong similarity in the localization pattern of Ecm11 and Zip1 prompted us to examine a possible physical association between them. We attempted to address the possibility by immunoprecipitaion. However, Zip1 was found to be highly unstable under native conditions in the meiotic whole cell extract, and the immunoprecipitation efficiency was extremely low, which kept us from testing the physical association of these proteins (data not shown).

**Figure 5:**
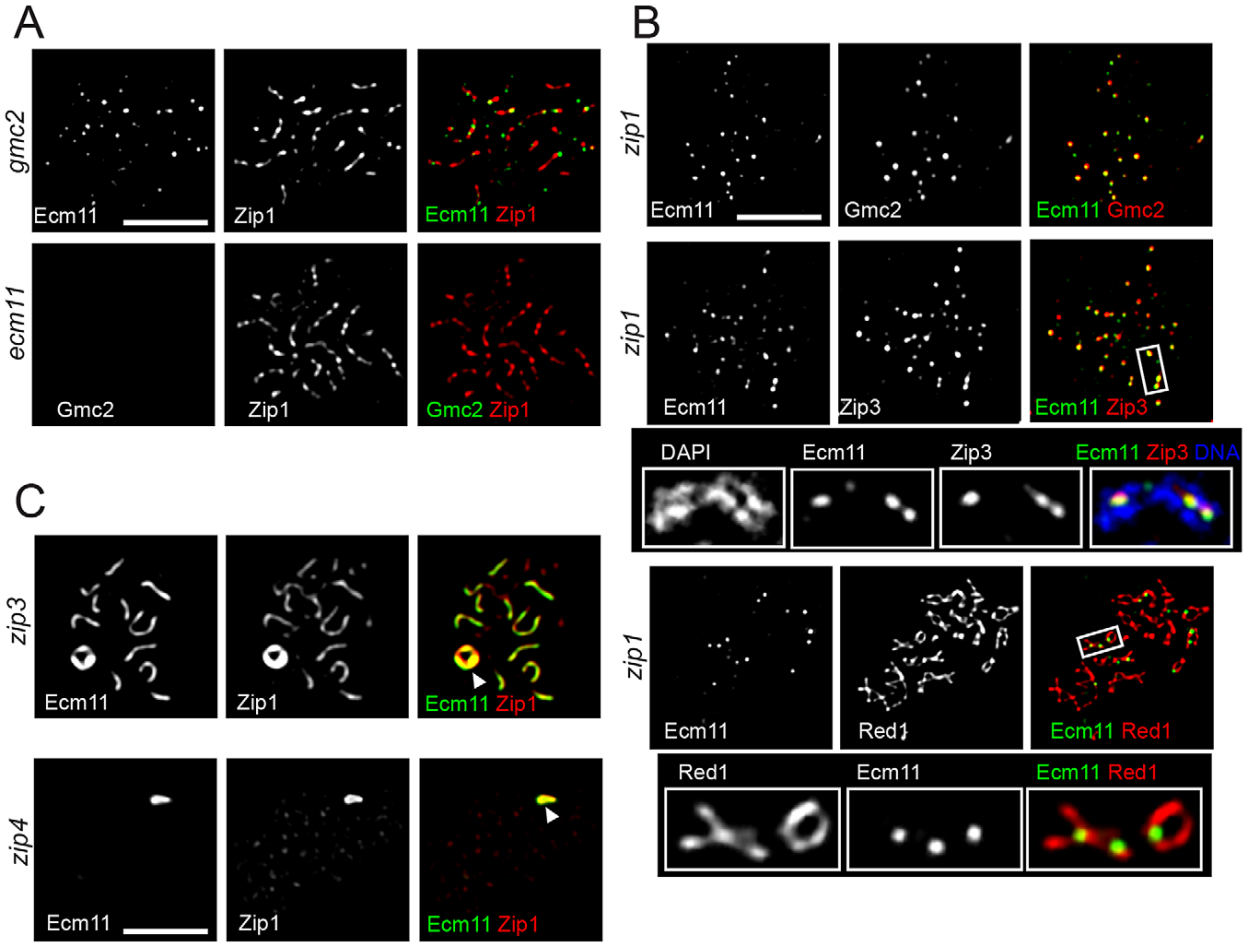
Genetic requirement for the chromosomal localization of Ecm11 and Gmc2. (A) Chromosomal localization of Ecm11 is compromised by the absence of Gmc2 while Ecm11 is indispensable for the localization of Gmc2. The *gmc2* mutant carrying *Ecm11-myc*, or the *ecm11* mutant carrying myc-Gmc2 was introduced into meiosis and the indicated proteins were identified by immunostaining. (B) Ecm11 and Gmc2 remain at the synapsis initiation sites in the absence of Zip1. Meiosis chromosomes were stained for the proteins indicated. The *zip1* mutant carrying *ECM11*-*FLAG* and *myc-GMC2* was used to examine the localization of Ecm11 and Gmc2. The *zip1* mutant carrying *ECM11*-*FLAG* and *ZIP3-myc* was used to examine Ecm11 and Zip3. The *zip1* mutant carrying *ECM11-myc* was used to examine Ecm11 and Red1. (C) Ecm11 is mislocalized to the polycomplex in the absence of a component of the synapsis initiation complex. Meiotic chromosomes of the *zip3* or *zip4* mutant carrying *ECM11-myc* were examined for the proteins indicated. White arrowheads indicate polycomplexes. A similar mislocalization was observed for Gmc2 as well (Figure S6B). Bar, 5 µm.

Next, the genetic requirement for the chromosomal localization of Ecm11 and Gmc2 was examined. Gmc2 localization to chromosomes was completely abolished in the absence of Ecm11, while an observable degree of Ecm11 remained localized to chromosomes in the absence of Gmc2 (Figure 5A). This chromosomal Ecm11 remains as foci, not exhibiting extensive colocalization with Zip3, with a 0.29 Pearson's correlation coefficient (median, n = 23) compared to 0.76 in the presence of Gmc2 (in the *zip1* mutant background where Ecm11 remains as foci, Figure S5B). These data highlight the importance of Gmc2 in the efficient targeting of Ecm11 to the synapsis initiation site.

In the absence of Zip1, both Ecm11 and Gmc2 still showed extensive colocalization, but remained as distinct foci without showing a line-like localization pattern (Figure 5B top, Figure S5B). Ecm11 colocalized with Zip3 between aligned chromosomes (Figure 5B middle, Figure S5B) and recruitment of Ecm11 to these sites depended on SIC components, Zip3 and Zip4 (Figure S6A). These sites tend to be in close proximity to the axial association sites visualized by localizing the Red1 protein, a component of meiotic chromosome axes (Figure 5B bottom). These results suggest the following three properties of the Ecm11 and Gmc2 proteins. First, they can recognize synapsis initiation sites independently of Zip1. Second, they are not components of the meiotic chromosome axis, since they are not localized to chromosome axes in the absence of Zip1. Third, the deposition of Ecm11 and Gmc2 along the central region between paired homologs requires Zip1.

In the absence of Zip3, initiation of synapsis becomes less efficient, leading to a decreased number of fully synapsed chromosomes with an elevated frequency of polycomplex formation. Ecm11 and Gmc2 were localized to both synapsed regions and the polycomplex in the *zip3* mutant, showing extensive colocalization with Zip1 (Figure 5C and Figure S6B). In the absence of Zip4, they appeared exclusively localized to the polycomplex along with Zip1. The chromosomal localization of Zip1 is almost completely abolished in the absence of Zip4, except at centromeres [8]. These observations are consistent with the idea that the confluent loading of Ecm11 and Gmc2 between paired homologs relies on Zip1. The absence of Ecm11 foci in either *zip3* or *zip4* mutant suggests that the recruitment of Ecm11 and Gmc2 to the synapsis initiation sites requires both Zip3 and Zip4, consistent with the observation that the recruitment of Ecm11 to axial association sites in the *zip1* mutant requires SIC components (Figure S6A).

The localization of Ecm11 in the absence of the initiation of meiotic recombination was examined next. In the *spo11* mutant, Ecm11 was primarily localized to the polycomplex along with Zip1 (Figure S6C). However, unlike Zip1, Ecm11 was not localized to centromeres (Figure S6C), consistent with the observation that the *ecm11* mutation has little effect on centromere coupling (Figure S3).

### SUMOylation at Lysine 5 of Ecm11 encourages the assembly of Zip1 specifically in the context of paired homologs

**Figure 6:**
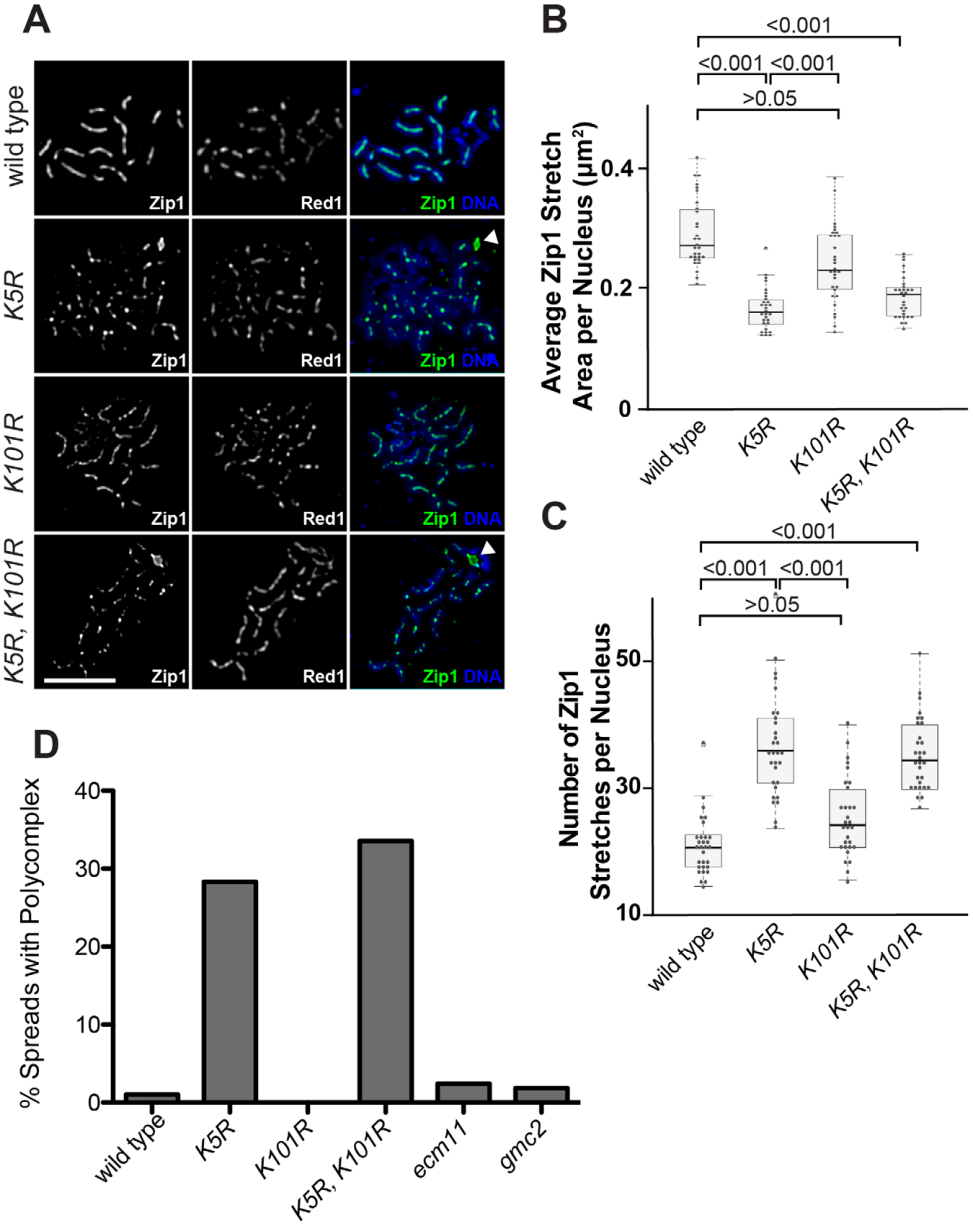
SUMOylation of Ecm11 at Lysine 5 is essential for facilitating the chromosomal assembly of Zip1. (A) The predominant role of Lysine 5 in facilitating the chromosomal assembly of Zip1. Meiotic chromosomes of wild type cells or *ecm11* mutants in which SUMOylation was compromised were examined for their Zip1 localization and also for Red1. Bar, 5 µm. (B, C) Quantitative analyses of Zip1 localization. See Figure legend of Figure 2B, 2C and also Results and Materials and Methods for details. (D) Polycomplex formation was elevated when Lysine 5 was mutated. Meiotic chromosomes carrying the mutations indicated were stained for the Zip1 protein and the fractions of chromosome spreads carrying a polycomplex were calculated. At least 100 chromosome spreads were counted for each strain.

To understand the physiological role of SUMOylation of Ecm11, mutations that partially or completely abolish the SUMOylation of Ecm11 were examined to observe their effect on Zip1 assembly by measuring the area of regions of continuous Zip1 staining (Zip1 stretches) and the number of Zip1 stretches per nucleus. We employed the K5R and K101R mutants, as well as the K5R K101R double mutant. The K5R mutant and K5R K101R double mutant exhibited a marked reduction in the average Zip1 stretch area and the number of stretches increased to levels comparable to that of the *ecm11* null mutant whereas K101R showed little effect (Figure 6A–6C and Figure S5C), suggesting a more critical role for the SUMOylation at K5 in facilitating the assembly of the interchromosomal Zip1 filament.

While the localization of Zip1 was being examined, we noticed that polycomplex formation was present ∼30% more frequently in the K5R mutant and the K5R K101R double mutants (Figure 6A and 6D). This is strikingly different from the *ecm11* or *gmc2* null mutant where polycomplex formation is rare. Taken together, these results suggest that chromosomal Zip1 assembly, as opposed to the polycomplex, is selectively compromised when the SUMOylation at K5 is prohibited, thus highlighting the role of SUMOylation in specifically facilitating the assembly of Zip1 at the proper location, between paired homologs.

## Discussion

### The Ecm11-Gmc2 complex is a novel SC component that facilitates the assembly of Zip1

Here we have identified two meiosis-specific proteins, Ecm11 and Gmc2, as novel components of the SC. They are likely to function in the central region of the SC rather than the lateral elements, since they show extensive colocalization with Zip1, a major component of the central region of the SC, and their localization is highly dependent on Zip1. In the absence of Zip1, Ecm11 and Gmc2 are not found on chromosome axes but are localized to the synapsis initiation sites.

Our observations indicate that Ecm11 and Gmc2 function as a complex. First, the *ecm11* and *gmc2* single mutants exhibit identical phenotypes. Zip1 assembly and crossing over are compromised to a similar level in both mutants. Second, they show extensive colocalization on meiotic chromosomes throughout meiotic prophase I. Third, the localization of Gmc2 is completely abolished without Ecm11. Fourth, the Ecm11 and Gmc2 proteins interact, as shown by Y2H and immunoprecipitation assays. Thus, the complex consisting of Ecm11 and Gmc2 is hereafter referred to as the E-G complex.

Our observations strongly argue for a function of the E-G complex in facilitating the assembly of Zip1 in the context of both chromosomes and the polycomplex. First, the E-G complex shows extensive colocalization with Zip3, a component of the SIC whose role is to initiate assembly of Zip1 filaments. Second, the E-G complex shows extensive colocalization with Zip1. Third, the absence of the E-G complex compromises the assembly of chromosomal Zip1 filaments. Fourth, in the *spo11* mutant background, polycomplex formation becomes dramatically reduced in the absence of the E-G complex.

We further provide evidence that the SUMOylation of the Ecm11 protein, especially at K5, is important for promoting the interchromosomal assembly of Zip1 filaments. In the SUMOylation-negative *ecm11* mutant, assembly of the chromosomal Zip1 was compromised substantially while polycomplex formation was not. Importantly, this phenotype is different from that of the *ecm11* null mutant in which the assembly of Zip1 is compromised in the context of both chromosomes and the polycomplex. These results suggest that the E-G complex is capable of facilitating the assembly of Zip1 without being SUMOylated, and that the SUMOylation helps specify the location of where Zip1 assembly is promoted. We cannot rule out the possibility that the pronounced polycomplex formation found in the K5R mutant is associated with a possible protein misfolding caused by the introduced mutation instead of lack of SUMO conjugation, although we do know the mutant proteins retain stability comparable to the wild type counterpart (Figure 3D).

**Figure 7:**
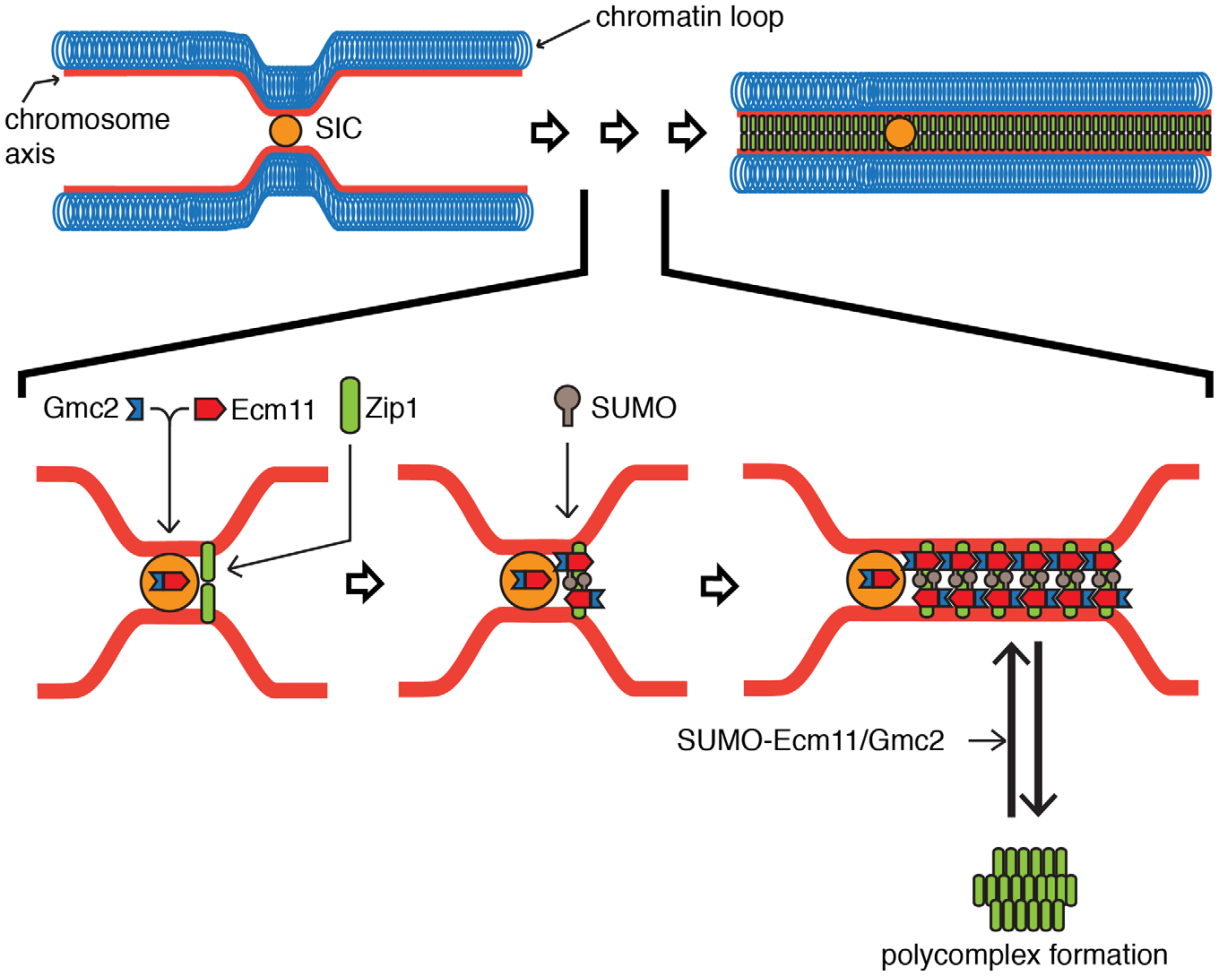
Proposed model for the function of the Ecm11-Gmc2 complex. The association of Ecm11 with Gmc2, forming the E-G complex (Ecm11-Gmc2), facilitates its loading at the synapsis initiation complex (SIC). In parallel, Zip1 is recruited to the SIC. The presence of the SIC and Zip1 with the E-G complex facilitates the SUMOylation of Ecm11. The SUMOylated E-G complex selectively promotes the chromosomal assembly of Zip1 while polycomplex formation is discouraged. See discussion for details.

Based on our results, we propose a mechanism of the E-G complex in facilitating the chromosomal assembly of Zip1 as the following steps (Figure 7). First, Ecm11 and Gmc2 form a complex (the E-G complex), which facilitates the recruitment of the E-G complex to a SIC. At the same time, the SIC, possibly along with the E-G complex, facilitates the initial assembly of Zip1. Second, the presence of Gmc2, the SIC and Zip1 strongly promotes SUMOylation of Ecm11, thus facilitating chromosomal assembly of Zip1 while discouraging polycomplex formation. The SUMOylation of Ecm11 almost completely requires Gmc2, and is also dependent, to a lesser extent, on Zip1, Zip2, Zip4 and Spo16. Third, the E-G complex stays with the assembled Zip1, possibly contributing to the stabilization of the assembled Zip1.

### Molecular function of the E-G complex

Based on these results, the E-G complex is likely to function through at least two different protein-protein interactions. First, the E-G complex can be recruited to the SIC in the absence of Zip1, suggesting that it interacts with at least one component of the SIC. Later, it acts together with Zip1 at locations where the SIC is absent. It is thus likely that the E-G complex interacts with Zip1 or other component(s) of the central region of the SC. Intriguingly, we show that Zip1 behavior in the *ecm11* null mutant is different to Zip1 behavior in the SUMO-negative mutant, highlighting the importance of SUMOylation in facilitating Zip1 assembly in a chromosomal context. One possibility is that the SUMOylation might promote/enhance the association between the lateral elements and transverse filament, making the structure of parallel lateral elements mediated by transverse filaments more rigid. In that sense, it is interesting that Red1, a component of the lateral element, has a SUMO-interacting motif (SIM) [27]. Given that the E-G complex is a component of the central region, the interaction between the E-G complex and Red1 through SUMO should contribute to the further stability of the interaction between the central region and the lateral elements of the SC. Red1 is also known to be SUMOylated, and Zip1 also has SIMs [24], [26], [27]. Previous work proposed that this potential interaction between Red1 and Zip1 contributes to the association between the central region and the lateral element [26], [27]. However, the SC was still established, although with slower kinetics, in the SUMO-negative *red1* mutant, suggesting there might be other interaction(s) stabilizing the SC. One such interaction could be between the E-G complex and Red1. It is also possible that the interaction between Zip1 SIM and SUMOylated E-G complex promotes SC formation by, for example, stabilizing the assembled Zip1. Further characterization will be able to address the role of SUMOylation of the E-G complex in SC formation at the molecular level.

### The E-G complex and the central element proteins

In this work, we have characterized the E-G complex as a facilitator for the assembly of the transverse filament (Zip1). Although we have not been successful in identifying orthologs of the Ecm11 or Gmc2 protein in other model organisms based on amino acid sequence similarity, proteins proposed to retain a similar function to the E-G complex have been reported in other model organisms. Proteins cytologically localized to the central element of the SC are proposed to facilitate assembly of the transverse filament by serving as stabilizing pillars [33]. These are SYCE1, SYCE2, SYCE3 and TEX12 in mice, and Corona in Drosophila [34]–[38]. In the absence of these proteins, SC formation is severely compromised or abolished. In the absence of Corona, not only is SC formation abrogated, but polycomplex formation is also abolished [38]. These apparent phenotypic similarities caused by the absence of the E-G complex and the central element proteins raise the possibility that the E-G complex might be functionally related to the central element proteins, possibly serving as a pillar to stabilize the transverse filament.

## Materials and Methods

### Strains and plasmids

Strains used are listed in Table S1 [39], [40]–[42]. Strains used in each Figure are summarized in Text S1. Gene deletions and C-terminus epitope tagging was performed using PCR-mediated gene replacement and tagging techniques as described previously [43]. N-terminus epitope tagging was performed as described previously [44]. Based on the kinetics of sporulation and spore viability, the functionality of the tagged proteins is comparable to that of untagged counterparts. *ecm11-K5R*, *-K101R*, -*K5N* and -*K101N* were created using the delitto perfetto method [45].

For yeast two-hybrid protein analyses, PCR-amplified *ECM11* and *GMC2* excluding the intron were cloned between the PvuII and NcoI sites of the pOAD and pOBD2 plasmids [46]. *GAL* sequences were fused to the 5′ end of each ORF.

### Cytology-based screening for genes encoding proteins associated with meiotic chromosomes

In a haploid strain carrying *MAT* **a** and *MATα*, and also carrying a *hop2* mutation (TBR569), a PCR-mediated method [43] was used to integrate the sequence encoding 13 copies of the myc antigen at the 3′-end of candidate genes whose transcripts are upregulated during early prophase I based on microarray analysis [47], [48]. The genes examined were: *CIK1*, *ECM11*, *ECM38*, *GMC2*, *HDR1*, *HSN1*, *IME4*, *MSN5*, *PIG1*, *RTG1*, *YBR231C*, *YDL012C*, *YDR018C*, *YDR374C*, *YGL081W*, *YHR202W*, *YLR387C*, *YMR147W*, *YSP3*. The tagged strains were introduced into meiosis and meiotic chromosomes were surface spread. Mouse anti-myc antibodies (Covance) were used to visualize candidate proteins.

### Measuring sporulation

Patches were made from a single colony of strains on YPD plates and incubated at 30°C overnight. The plates were replica plated onto sporulation medium and sporulation was examined at the indicated time points. For each strain, spore formation was measured in three independent experiments, with at least 300 cells scored in each experiment.

### Cytology

Meiotic chromosomes were surface spread, and immunostaining was carried out as described previously [42]. Rabbit and mouse anti-Red1 antibodies were used at 1∶500 and 1∶1000 dilutions respectively [49]. Rabbit anti-Zip1 antibodies were used at 1∶300 dilution [3]. Mouse and rabbit anti-myc antibody were used at 1∶300 dilution (Covance). Mouse anti-FLAG antibody was used at 1∶200 dilution (Sigma). Images were captured using the Deltavision IX70 system (Applied Precision), and *softWoRx* imaging software. Images were processed by deconvolution using the constrained iterative deconvolution algorithm within *softWoRx*, and appropriate consecutive deconvolved z-slices were projected together to form the final processed image.

### Quantitative evaluation of Zip1 localization

Zip1 distribution was quantified using various tools within *softWoRx*. Projected spread images of pachytene nuclei (determined by presence of condensed chromatin by DAPI staining) were used to obtain signal intensity values for the spread-containing region of the image in the green channel. The 90th percentile value for signal intensity was calculated and utilised as a threshold value for the ‘Polygon Finder’ tool within *softWoRx*, which identified continuous regions of Zip1 localization or ‘stretches’. The region incorporating the whole spread area, and a threshold perimeter of 10 µm for polygons, was specified and polygons were calculated. Polygons identified outside the DAPI-stained area were manually de-selected and not used in the data. Polygon number and area were recorded and used to evaluate Zip1 distribution. At least 20 spread projections were analysed per strain.

### Protein colocalization analysis

Projected images were selected and analysed for colocalization between channels using the colocalization tool within *softWoRx*, which calculated Pearson's correlation coefficient to represent the degree of colocalization. The region of the image used for analysis was determined by DAPI staining. At least 10 spreads were analysed for each pair of proteins.

### Immunoprecipitation

Native whole cell extracts (WCE) were prepared using 50 ml sporulating culture. Pelleted cells were resuspended in 400 µl of lysis buffer (1 mM DTT, 0.05% Igepal CA-630, 200 mM NaCl, 10 mM EDTA, 10% Glycerol, 50 mM Tris-HCl, pH 8.0) containing protease inhibitors (1 mM PMSF and 1× protease inhibitor, EDTA-free (Roche)). Cells were lysed by beating six times for 20 seconds each in the presence of zirconia/silica beads at 4°C. Anti-FLAG or anti-myc antibody was incubated with WCE at 1∶125 dilution. Bound proteins were retrieved using protein G-coated Dynabeads (Invitrogen). Beads were washed, then bound proteins were eluted by SDS and used for Western blotting. In parallel, after the wash stage, the immunoprecipitates were treated with 0.125 unit/µl Benzonase (Merck) in lysis buffer without EDTA, supplemented with 2.5 mM MgCl_2_ and incubated for 30 min at 4°C. This treatment was enough to completely digest 3.75 µg of plasmid DNA (no trace of DNA was found, examined by agarose gel electrophoresis). The immunoprecipitates were subjected to Western blotting.

### Statistics

Statistical analysis was undertaken using InStat3 and Prism software (www.graphpad.com). Significance testing was done using the Kruskal-Wallis (nonparametric ANOVA) test with Dunn's multiple comparisions test. Graphs for Zip1 stretch number and area were drawn using the beeswarm and boxplot overlay packages in the R Project for statistical computing (Bioconductor).

### Other techniques

Denatured protein extracts and Western blotting were done as described before [25]. Mouse and rabbit anti-myc antibodies were used at 1∶2000 and 1∶1000 dilutions respectively (Covance). Mouse anti-FLAG antibody was used at 1∶1000 dilution (Sigma). The physical recombination assay was done as described before [42], [50]. For quantitation shown in Figure 1C, the signals from bands corresponding to linear dimer and trimer were measured and expressed as the percentage of the total signal (linear monomer, dimer and trimer).

## Supporting Information

Figure S1 *ecm11* and *gmc2* mutants do not exhibit calcofluor sensitivity. Wild type, *ecm11* and *gmc2* mutants were serially diluted (5-fold dilutions) and placed on complete medium containing various concentrations of calcofluor white as indicated.
(TIF)

Figure S2 *ecm11* and *gmc2* mutations do not show a synergistic effect in sporulation. Diploid cells were introduced into meiosis and spore formation was examined at indicated time points. Error bars represent SEM.
(TIF)

Figure S3 Centromere coupling occurs normally in the absence of Ecm11 or Gmc2. *spo11 ndt80 CTF19-myc* diploid cells with indicated mutations were introduced into meiosis, and spread chromosomes were stained for Ctf19 (centromere marker) and Red1. The number of Ctf19 foci per spread nucleus was counted. Bar, 5 µm.
(TIF)

Figure S4 Analyses of Ecm11 SUMOylation. (A–C) Western blot images shown in Figure 3 are presented along with the corresponding Ponceau S staining images. (A), Figure 3A; (B), Figure 3B; (C), Figure 3D. (D) Lysine to Asparagine mutants exhibit a similar SUMOylation defect to the Lysine to Arginine mutants. The *ndt80* diploid strain carrying wild type *ECM11-myc* or its mutated derivatives, *ecm11-K5N*, *ecm11-K101N* or *ecm11-K5N*, *K101N* was introduced into meiosis. The Ecm11 protein was detected using anti-myc antibody by Western blotting. (E) Ecm11 is SUMOylated. Whole cell extract (WCE) obtained from cells carrying *FLAG-SMT3 ECM11-myc* was immunoprecipitated using anti-myc antibodies. The immunoprecipitates were subjected to Western blotting using anti-FLAG and anti-myc antibodies. Native, the whole cell extract prepared using the conditions supporting the native structure of proteins. TCA, the whole cell extract prepared using TCA, which denatures proteins. * indicates the location of the immunoglobulin chain migrating around the same position as the second band of the modified forms of Ecm11. ** indicates the location of possible degradation products of Ecm11.
(TIF)

Figure S5 The relationship between the protein localization of Ecm11, Gmc2 and the SC components and the importance of SUMOylation of Ecm11 in chromosomal assembly of Zip1. (A) Pearson's correlation coefficients of a pair of proteins indicated were calculated in the wild type background. (B) Pearson's correlation coefficients of a pair of proteins indicated were calculated in the *zip1* background. (C) Quantitative analysis of the Zip1 localization in the strains indicated. Measurement was done as in Figure 2. One dot represents one chromosome spread through (A–C).
(TIF)

Figure S6 The localization of Emc11 and Gmc2 in various meiotic mutants. Meiotic chromosomes of the *zip1*, *zip1 zip3* or *zip1 zip4* mutants (A), the *zip3* or *zip4* (B) and the *spo11* mutant (C) were examined for the proteins indicated. In (B) and (C), white arrowheads indicate the polycomplex. Bar, 5 µm.
(TIF)

Table S1 List of strains used in this study.
(PDF)

Text S1 Supporting Materials and Methods.
(PDF)
