# Supplementary figures and images for: The Ecm11-Gmc2 Complex Promotes Synaptonemal Complex Formation through Assembly of Transverse Filaments in Budding Yeast
Source: PLoS Genet. 2013 Jan 10;9(1):e1003194. doi: 10.1371/journal.pgen.1003194 (PMC3542071; doi:10.1371/journal.pgen.1003194)

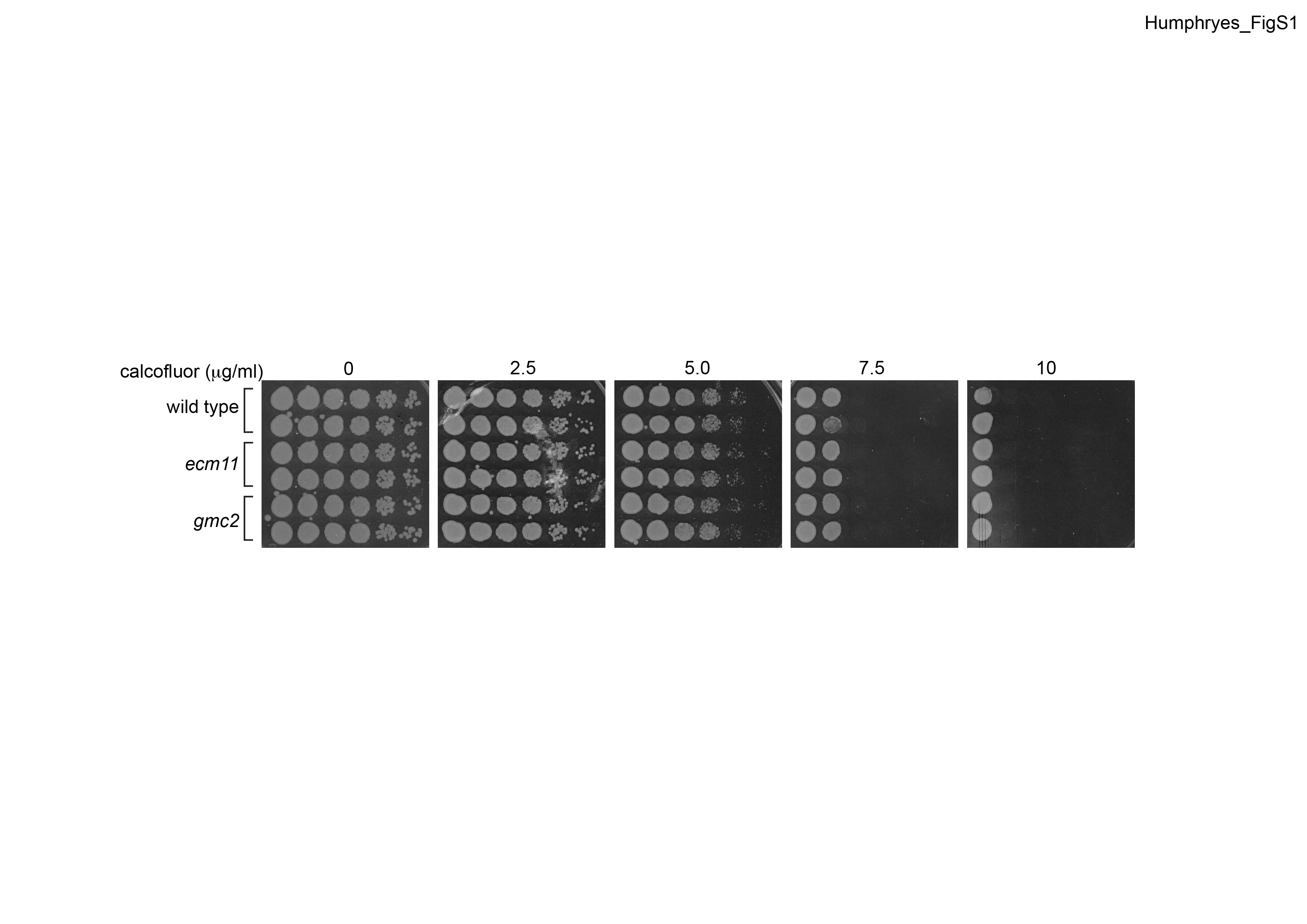

Supplement: Figure S1 — ecm11 and gmc2 mutants do not exhibit calcofluor sensitivity. Wild type, ecm11 and gmc2 mutants were serially diluted (5-fold dilutions) and placed on complete medium containing various concentrations of calcofluor white as indicated. (TIF) [file pgen.1003194.s001.tif]

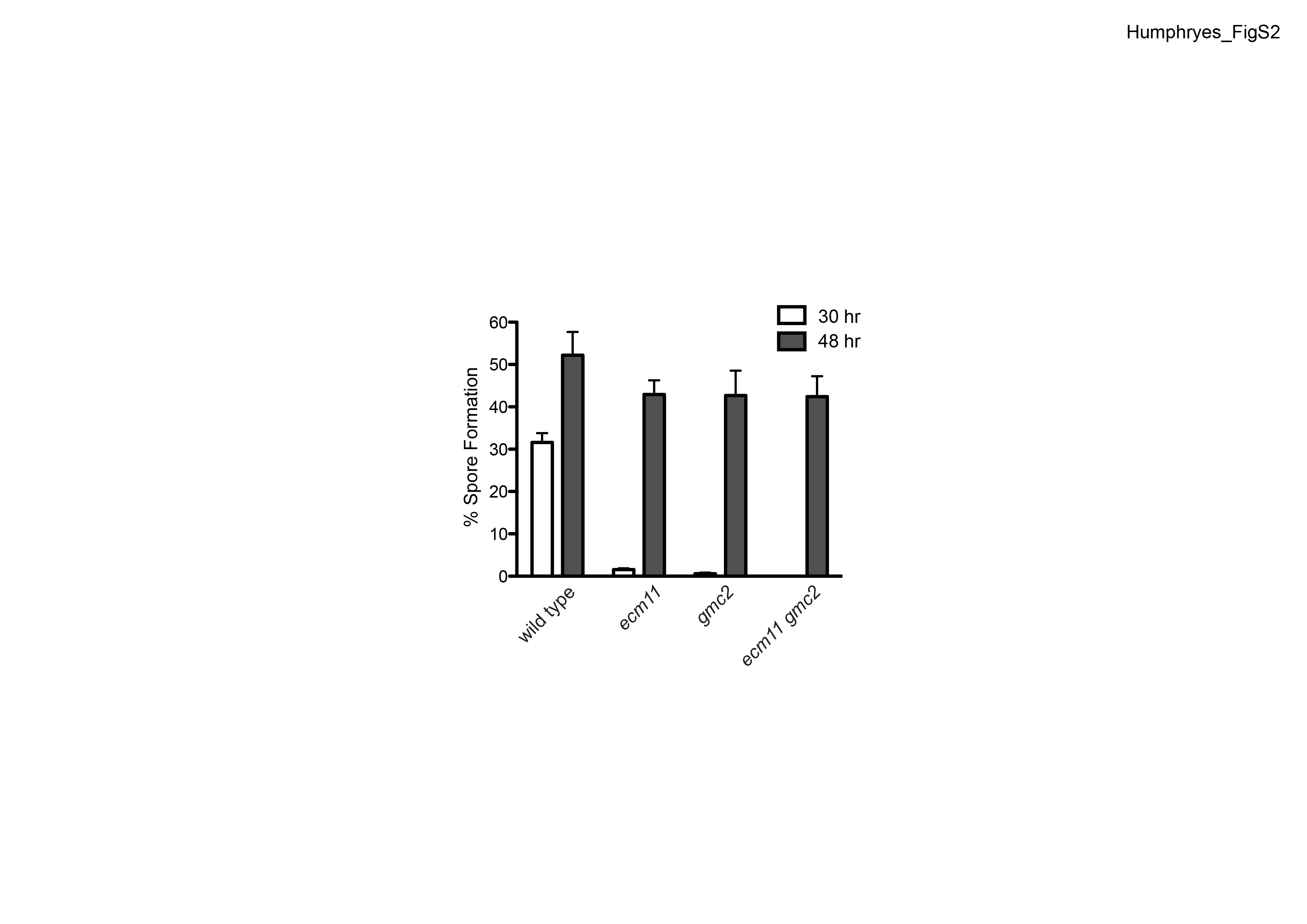

Supplement: Figure S2 — ecm11 and gmc2 mutations do not show a synergistic effect in sporulation. Diploid cells were introduced into meiosis and spore formation was examined at indicated time points. Error bars represent SEM. (TIF) [file pgen.1003194.s002.tif]

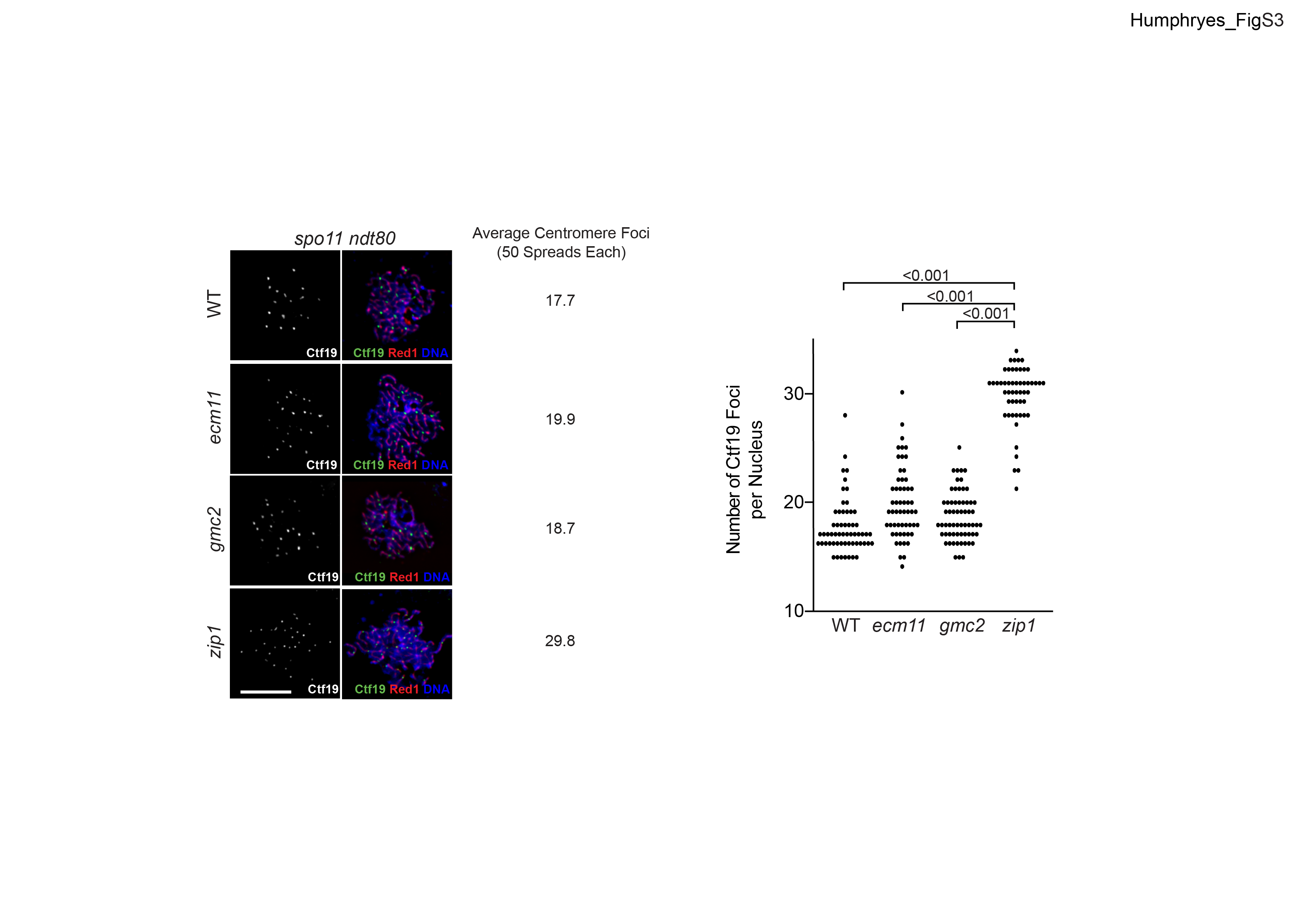

Supplement: Figure S3 — Centromere coupling occurs normally in the absence of Ecm11 or Gmc2. spo11 ndt80 CTF19-myc diploid cells with indicated mutations were introduced into meiosis, and spread chromosomes were stained for Ctf19 (centromere marker) and Red1. The number of Ctf19 foci per spread nucleus was counted. Bar, 5 µm. (TIF) [file pgen.1003194.s003.tif]

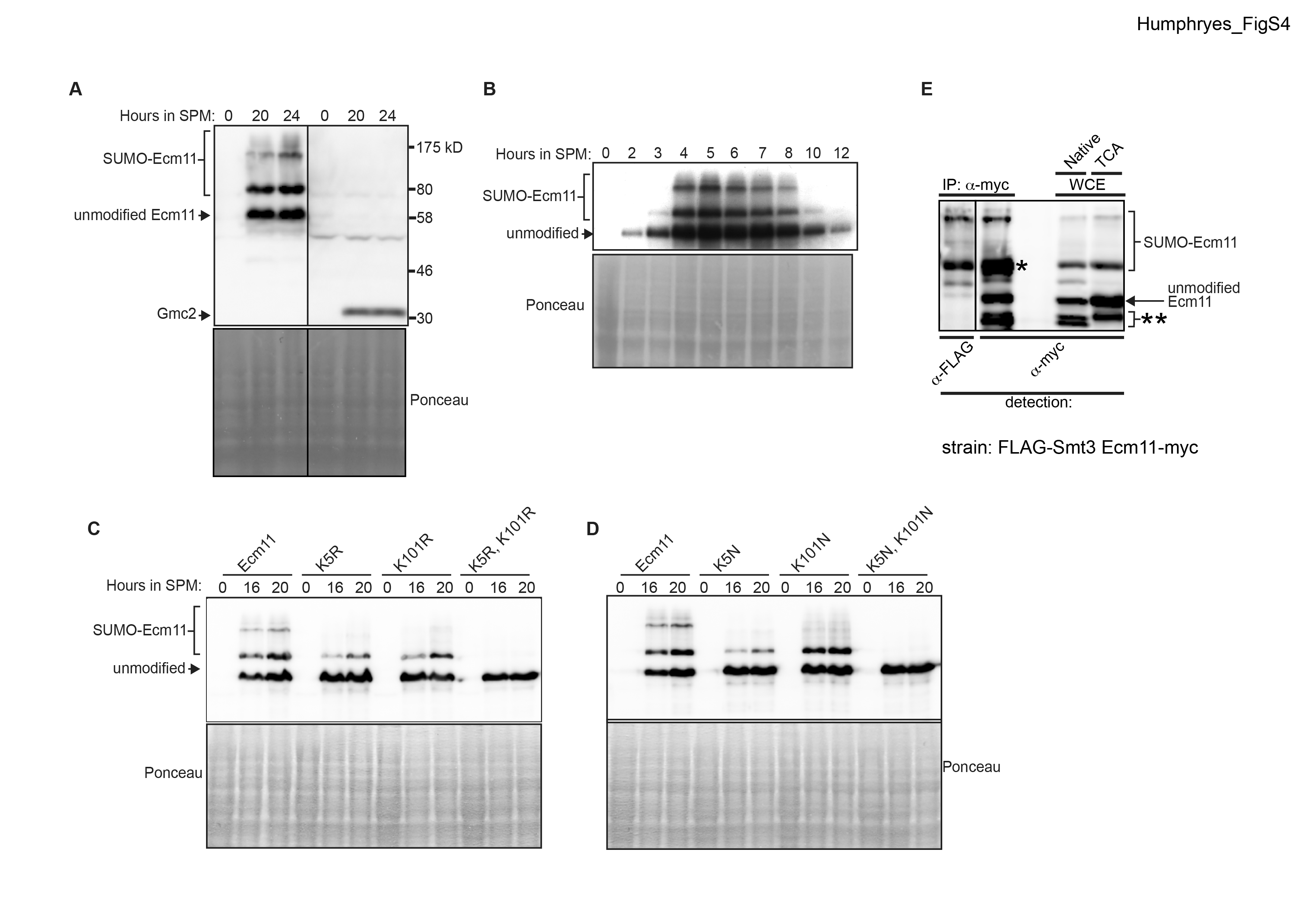

Supplement: Figure S4 — Analyses of Ecm11 SUMOylation. (A–C) Western blot images shown in Figure 3 are presented along with the corresponding Ponceau S staining images. (A), Figure 3A; (B), Figure 3B; (C), Figure 3D. (D) Lysine to Asparagine mutants exhibit a similar SUMOylation defect to the Lysine to Arginine mutants. The ndt80 diploid strain carrying wild type ECM11-myc or its mutated derivatives, ecm11-K5N, ecm11-K101N or ecm11-K5N, K101N was introduced into meiosis. The Ecm11 protein was detected using anti-myc antibody by Western blotting. (E) Ecm11 is SUMOylated. Whole cell extract (WCE) obtained from cells carrying FLAG-SMT3 ECM11-myc was immunoprecipitated using anti-myc antibodies. The immunoprecipitates were subjected to Western blotting using anti-FLAG and anti-myc antibodies. Native, the whole cell extract prepared using the conditions supporting the native structure of proteins. TCA, the whole cell extract prepared using TCA, which denatures proteins. * indicates the location of the immunoglobulin chain migrating around the same position as the second band of the modified forms of Ecm11. ** indicates the location of possible degradation products of Ecm11. (TIF) [file pgen.1003194.s004.tif]

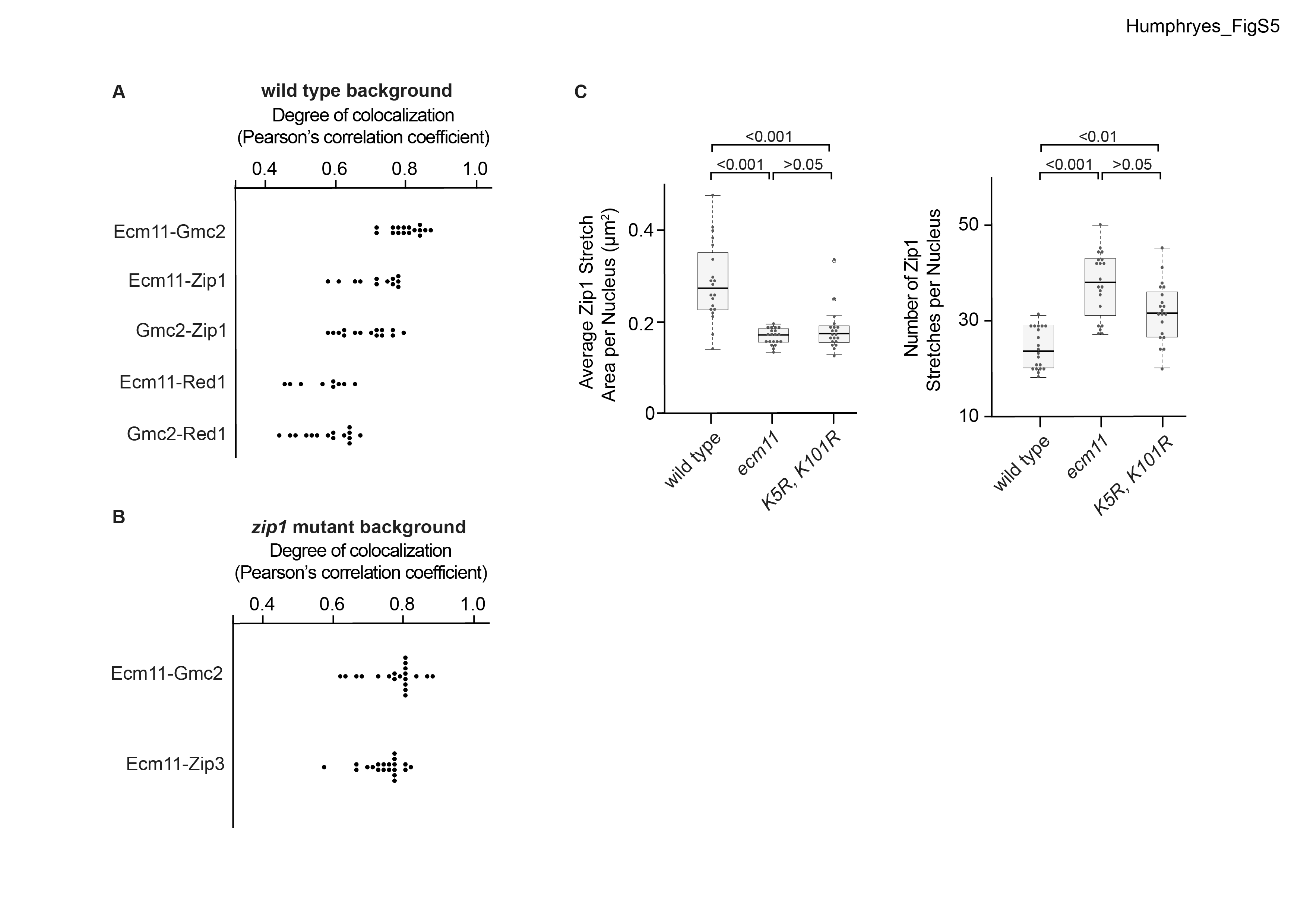

Supplement: Figure S5 — The relationship between the protein localization of Ecm11, Gmc2 and the SC components and the importance of SUMOylation of Ecm11 in chromosomal assembly of Zip1. (A) Pearson's correlation coefficients of a pair of proteins indicated were calculated in the wild type background. (B) Pearson's correlation coefficients of a pair of proteins indicated were calculated in the zip1 background. (C) Quantitative analysis of the Zip1 localization in the strains indicated. Measurement was done as in Figure 2. One dot represents one chromosome spread through (A–C). (TIF) [file pgen.1003194.s005.tif]

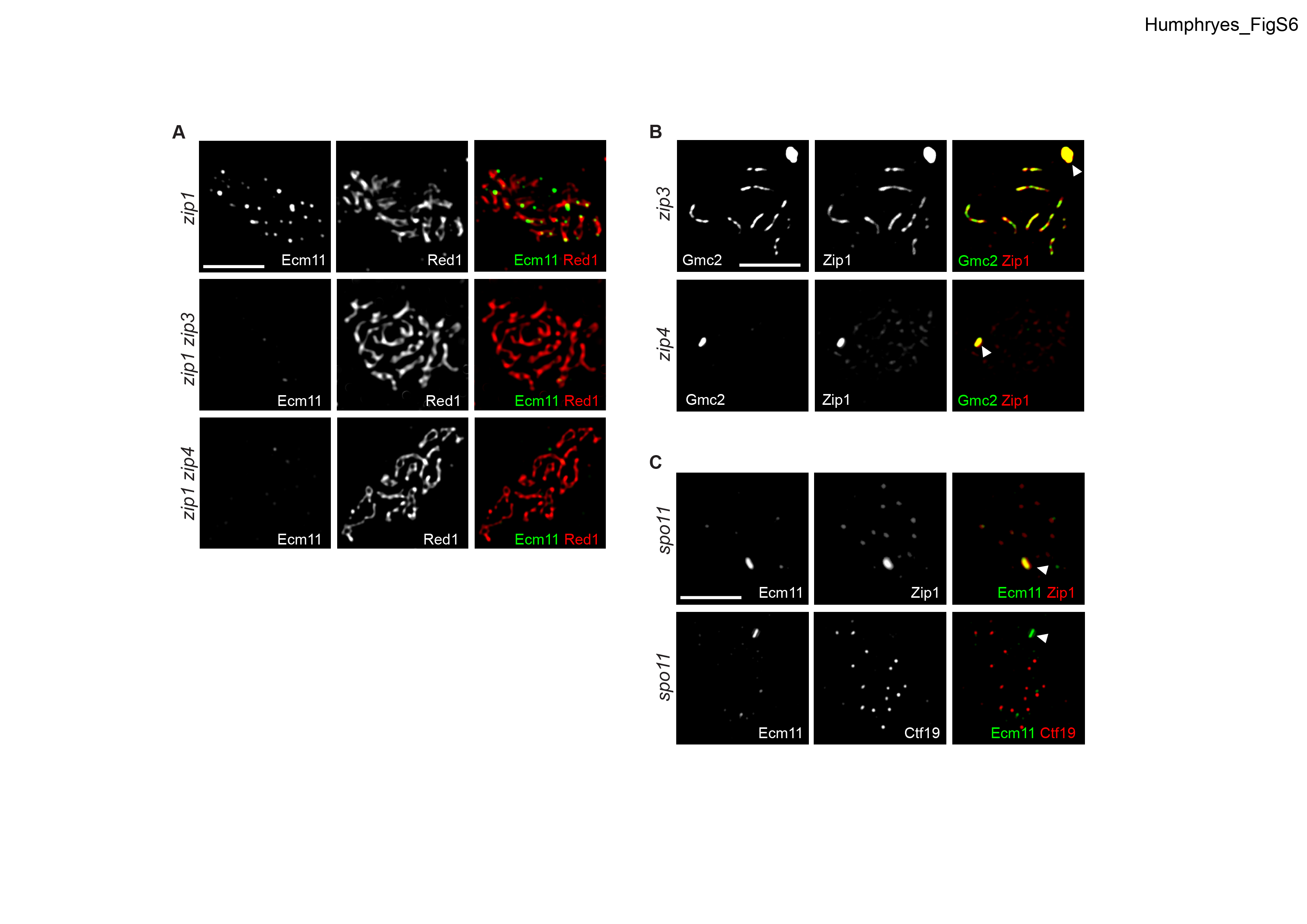

Supplement: Figure S6 — The localization of Emc11 and Gmc2 in various meiotic mutants. Meiotic chromosomes of the zip1, zip1 zip3 or zip1 zip4 mutants (A), the zip3 or zip4 (B) and the spo11 mutant (C) were examined for the proteins indicated. In (B) and (C), white arrowheads indicate the polycomplex. Bar, 5 µm. (TIF) [file pgen.1003194.s006.tif]
