# Supplementary material for: The Ecm11-Gmc2 Complex Promotes Synaptonemal Complex Formation through Assembly of Transverse Filaments in Budding Yeast
Source: PLoS Genet. 2013 Jan 10;9(1):e1003194. doi: 10.1371/journal.pgen.1003194 (PMC3542071; doi:10.1371/journal.pgen.1003194)
Supplement: Table S1 — List of strains used in this study. (PDF) [file pgen.1003194.s007.pdf]

Table S1. Yeast strains

Strain	Genotype
TBR2065	*MAT***a** *leu2-3, 112 his4-260 ura3-1 ade2-1 thr1-4 trp1-289 lys2*<br>---<br>*MAT*α *leu2-3, 112 his4-260 ura3-1 ade2-1 thr1-4 trp1-289 lys2*
TBR4246	TBR2065 but homozygous *ecm11::KAN*
TBR4327	TBR2065 but homozygous *gmc2::NAT*
TBR309	TBR2065 but homozygous *spo11::ADE2*
TBR5150	TBR2065 but homozygous *spo11::ADE2 ecm11::KAN*
TBR5148	TBR2065 but homozygous *spo11::ADE2 gmc2::NAT*
TBR2288[a]	*MAT***a** *leu2-3, 112 his4-260 ura3-1 ade2-1 thr1-4 trp1-289 lys2*<br>---<br>*MAT*α *his4-260 ura3-1 ade2-1 thr1-4 trp1-289 lys2 cup1Δ leu2::CUP1 arg4-8 TRP1*
TBR4282[a]	TBR2288 but homozygous *ecm11::clonNAT*
TBR4326[a]	TBR2288 but homozygous *gmc2::clonNAT*
TBR4757	TBR2065 but homozygous *ecm11::KAN gmc2::clonNAT*
TBR5296	TBR2065 but homozygous *ECM11-13myc-KAN ndt80::LEU2*
TBR6014	TBR2065 but homozygous *3myc-GMC2 ndt80::LEU2*
TBR5773	*MAT***a** *ho::hisG leu2::hisG ura3(delta Sma-Pst) HIS4::LEU2-(NBam)* *ECM11-13Myc-KAN*

MATα ho::hisG leu2::hisG ura3(delta Sma-Pst) his4X::LEU2-(NBam)-URA3 ECM11-13Myc-KAN

TBR5850 TBR2065 but homozygous *ECM11-13myc-KAN SMT3-3FLAG-TRP1 ndt80::LEU2*

TBR4321 TBR2065 but homozygous *ECM11-13myc-KAN*

TBR5632 TBR2065 but homozygous *ecm11_K5R-13myc-KAN*

TBR5634 TBR2065 but homozygous *ecm11_K101R-13myc-KAN*

TBR5511 TBR2065 but homozygous *ecm11_K5R_K101R-13myc-KAN*

TBR6085 TBR2065 but homozygous *ECM11-13myc-KAN gmc2::clonNAT ndt80::LEU2*

TBR5311 TBR2065 but homozygous *ECM11-13myc-KAN spo11::ADE2 ndt80::LEU2*

TBR6090 TBR2065 but homozygous *ECM11-13myc-KAN zip1::LEU2 ndt80::LEU2*

TBR6088 TBR2065 but homozygous *ECM11-13myc-KAN zip3::URA3 ndt80::LEU2*

TBR5848 TBR2065 but homozygous *ECM11-13myc-KAN spo16::NAT ndt80::LEU2*

TBR5734 TBR2065 but homozygous *ECM11-13myc-KAN zip2::LEU2 ndt80::LEU2*

TBR5736 TBR2065 but homozygous *ECM11-13myc-KAN zip4::URA3 ndt80::LEU2*

TBR5495 TBR2065 but homozygous *ECM11-3FLAG-KAN 3myc-GMC2*

TBR4648 TBR2065 but homozygous *3myc-GMC2*

TBR5977 TBR2065 but homozygous *ECM11-3FLAG-KAN ZIP3-3myc*

PJ69-4A	*MAT***a** t*rp1-901 leu2-3,112 ura3-52 his3-200 gal4Δ gal8Δ LYS2::GAL1-HIS3 GAL2-ADE2 met2::GAL7-lacZ*
TBR6549	TBR2065 but homozygous *ECM11-3FLAG-KAN 3myc-GMC2 ndt80::LEU2*
TBR6595	TBR2065 but homozygous *ECM11-3FLAG-KAN ndt80::LEU2*
TBR5305	TBR2065 but homozygous *ECM11-13myc-KAN gmc2::clonNAT*
TBR5309	TBR2065 but homozygous *3myc-GMC2 ecm11::KAN*
TBR6570	TBR2065 but homozygous *ECM11-3FLAG-KAN 3myc-GMC2 zip1::LEU2 ndt80::LEU2*
TBR6548	TBR2065 but homozygous *ECM11-3FLAG-KAN ZIP3-3myc zip1::LEU2 ndt80::LEU2*
TBR6070	TBR2065 but homozygous *ECM11-13myc-KAN zip1::LEU2*
TBR6078	TBR2065 but homozygous *ECM11-13myc-KAN zip3::URA3*
TBR5994	TBR2065 but homozygous *ECM11-13myc-KAN zip4::URA3*
TBR7313	TBR2065 but homozygous *ECM11-13myc-KAN zip1::LEU2 zip3::URA3*
TBR7314	TBR2065 but homozygous *ECM11-13myc-KAN zip1::LEU2 zip4::URA3*
TBR6306	TBR2065 but homozygous *ecm11_K5R 3myc-GMC2*
TBR6304	TBR2065 but homozygous *ecm11_K101R 3myc-GMC2*
TBR6305	TBR2065 but homozygous *ecm11_K5R_K101R 3myc-GMC2*
TBR4637	TBR2065 but homozygous *CTF19-13myc-KAN spo11::ADE2 ndt80::LEU2*

TBR4733	TBR2065 but homozygous *CTF19-13myc-KAN spo11::ADE2 ndt80::LEU2 ecm11::clonNAT*
TBR5756	TBR2065 but homozygous *CTF19-13myc::KAN ndt80::LEU2 spo11::ADE2 gmc2::cloNAT*
TBR6140	TBR2065 but homozygous *CTF19-13myc::KAN spo11::ADE2 ndt80::LEU2 zip1::LEU2*
TBR7302	TBR2065 but homozygous *CTF19-13myc::KAN spo11::ADE2 ECM11-3FLAG-KAN*
TBR5631	TBR2065 but homozygous *ecm11_K5N-13myc-KAN*
TBR5507	TBR2065 but homozygous *ecm11_K101N-13myc-KAN*
TBR5504	TBR2065 but homozygous *ecm11_K5N_K101N-13myc-KAN*
TBR5913	TBR2065 but homozygous *3myc-GMC2 zip3::URA3*
TBR5998	TBR2065 but homozygous *3myc-GMC2 zip4::URA3*

[a]One of chromosome III is circular [42]. All the strains except TBR5773 and PJ69-4A are isogenic with BR1919-8B [39]. TBR5773 is SK1 strain [41]. PJ69-4A was described previously [40].
