# Supplementary material for: The Ecm11-Gmc2 Complex Promotes Synaptonemal Complex Formation through Assembly of Transverse Filaments in Budding Yeast
Source: PLoS Genet. 2013 Jan 10;9(1):e1003194. doi: 10.1371/journal.pgen.1003194 (PMC3542071; doi:10.1371/journal.pgen.1003194)
Supplement: Text S1 — Supporting Materials and Methods. (PDF) [file pgen.1003194.s008.pdf]

Supporting Materials and Methods

Strains used in each Figure

Strains used in each Figure are as follows. Figure 1A, wild type (TBR2065), *ecm11* (TBR4246), *gmc2* (TBR4327), *spo11* (TBR309), *spo11 ecm11* (TBR5148) and *spo11 gmc2* (TBR5150). Figure 1BC, circular linear, wild type (TBR2288), *ecm11* (TBR4282), *gmc2* (TBR4326). Figure 2, wild type (TBR2065), *ecm11* (TBR4246), *gmc2* (TBR4327), *ecm11 gmc2* (TBR4757), *spo11* (TBR309), *spo11 ecm11* (TBR5150) and *spo11 gmc2* (TBR5148). Figure 3A, *ECM11-13myc ndt80* (TBR5296), *3myc-GMC2 ndt80* (TBR6014). Figure 3B, *ECM11-13myc* (TBR5773). Figure 3C, *3FLAG-SMT3 ECM11-13myc ndt80* (TBR5850), *ECM11-13myc ndt80* (TBR5296). Figure 3D, *ECM11-13myc* (TBR4321), *ecm11-K5R-13myc* (TBR5632), *ecm11-K101R-13myc* (TBR5634), *ecm11-K5R, K101R-13myc* (TBR5511). Figure 3E, *ECM11-13myc gmc2 ndt80* (TBR6085), *ECM11-13myc spo11 ndt80* (TBR5311), *ECM11-13myc zip1 ndt80* (TBR6090), *ECM11-13myc zip3* (TBR6088), *ECM11-13myc spo16* (TBR5848), *ECM11-13myc zip2* (TBR5734) and *ECM11-13myc zip4* (TBR5736). Figure 4A, *ECM11-3FLAG 3myc-GMC2* (TBR5495). Figure 4B, *ECM11-13myc* (TBR4321), *3myc-GMC2* (TBR4648). Figure 4C, *ECM11-3FLAG ZIP3-3myc* (TBR5977). Figure 4D, PJ69-4A. Figure 4E, *ECM11-3FLAG 3myc-GMC2 ndt80* (TBR6549), *ECM11-3FLAG ndt80* (TBR6595), *3myc-GMC2 ndt80* (TBR6014). Figure 5A, *ECM11-13myc gmc2* (TBR5305), *3myc-GMC2 ecm11* (TBR5309). Figure 5B, *ECM11-3FLAG, 3myc-GMC2 zip1 ndt80* (TBR6570), *ECM11-3FLAG ZIP3-3myc ndt80* (TBR6548), *ECM11-13myc zip1* (TBR6070). Figure 5C, *ECM11-13myc zip3* (TBR6078), *ECM11-13myc zip4* (TBR5994). Figure 6, wild type (TBR2065), *ecm11-K5R* (TBR6306), *ecm11-K101R* (TBR6304), *ecm11-K5R, K101R* (TBR6305), *ecm11* (TBR4246), *gmc2* (TBR4327). Figure S1, wild type (TBR2065), *ecm11* (TBR4246), *gmc2* (TBR4327).

Figure S2, wild type (TBR2065), *ecm11* (TBR4246), *gmc2* (TBR4327) and *ecm11 gmc2* (TBR4757). Figure S3, *spo11 ndt80 CTF19-13myc* (TBR4637), *spo11 ndt80 CTF19-13myc ecm11* (TBR4733), *spo11 ndt80 CTF19-13myc gmc2* (TBR5756) and *spo11 ndt80 CTF19-13myc zip1* (TBR6140). Figure S4A *ECM11-13myc ndt80* (TBR5296), *3myc-GMC2 ndt80* (TBR6014). Figure S4B, *ECM11-13myc* (TBR5773). Figure S4C, *ECM11-13myc* (TBR4321), *ecm11-K5R-13myc* (TBR5632), *ecm11-K101R-13myc* (TBR5634), *ecm11-K5R, K101R-13myc* (TBR5511). Figure S4D, *ECM11-13myc* (TBR4321), *ecm11-K5N-13myc* (TBR5631), *ecm11-K101N-13myc* (TBR5507), *ecm11-K5N, K101N-13myc* (TBR5504). Figure S5A, *ECM11-3FLAG, 3myc-GMC2 ndt80* (TBR6549). Figure S5B, *3myc-GMC2 zip3* (TBR5913), *3myc-GMC2 zip4* (TBR5998). Figure S5C, wild type (TBR2065), ecm11 (TBR4246) and *ecm11-K5R, K101R* (TBR6305). Figure S6A, *ECM11-13myc zip1* (TBR6070), *ECM11-13myc zip1 zip3* (TBR7313) and *ECM11-13myc zip1 zip4* (TBR7314). Figure S6B, *3myc-GMC2 zip3* (TBR5913), *3myc-GMC2 zip4* (TBR5998). Figure S6C, *CTF19-13myc ECM11-3FLAG spo11* (TBR7302).
